# The Adipose‐Sympathetic Nerve Crosstalk: FSTL1 as an Adipocyte‐Derived Neurotrophic Factor for WAT Browning

**DOI:** 10.1002/advs.77433

**Published:** 2026-08-31

**Authors:** Xiao‐Wei Jia, Jia‐Xin Liu, Rui‐Ping Wang, Xue‐Qin Wu, Yao Che, Mu‐Chen Wu, Hong‐Wei Shang, Xin Lu, Tao Lu, Lu‐Lu Wang, Chun‐Yan Jiang, Dong‐Liang Fang, Chun Yang, Yan Gao

**Affiliations:** ^1^ Department of Human Anatomy School of Basic Medical Sciences Capital Medical University Beijing China; ^2^ Department of National Experimental Center for Basic Medical Teaching School of Basic Medical Sciences Capital Medical University Beijing China; ^3^ Sports Medicine Department Beijing Jishuitan Hospital Capital Medical University Beijing China; ^4^ Key Laboratory of Major Diseases in Children Ministry of Education Beijing China

**Keywords:** browning, follistatin‐like 1, innervation, obesity

## Abstract

Follistatin‐like 1 (FSTL1) has been considered a vital adipokine. Several lines of evidence have shown that FSTL1 is essential for white adipose tissue (WAT) adipogenesis, brown adipose tissue involution, and thermogenesis. However, the roles of FSTL1 in WAT browning and its underlying mechanisms are not fully understood. Our data show that FSTL1 overexpression induces WAT browning, enhances energy metabolism, and promotes glucose uptake in male mice at ambient room temperature. Overexpression of FSTL1 specifically in WAT prevents metabolic dysfunction in mice with diet‐induced obesity. Mice with FSTL1 deficiency exhibit cold intolerance and are prone to obesity. FSTL1 cannot directly induce adipocyte browning in vitro. FSTL1 promotes sympathetic innervation both in vivo and in vitro. Furthermore, sympathetic denervation in adipose tissue abrogates FSTL1‐induced browning, enhancement of energy metabolism, and regional glucose uptake. Notably, FSTL1 is endocytosed by sympathetic neurons primarily via tropomyosin‐related kinase B (TrkB) in vitro. Blocking TrkB suppresses WAT browning and sympathetic innervation induced by FSTL1 overexpression in adipose tissue. Our findings shed new light on the role of FSTL1 in WAT browning and identify FSTL1 as an adipose‐derived ‘neurotrophic factor’ adipokine.

## Graphical abstract

As a novel adipose‐derived neurotrophic factor, Follistatin‐like 1 is endocytosed by sympathetic neurons primarily via tropomyosin‐related kinase B. This process promotes sympathetic innervation in adipose tissue and norepinephrine release, leading to white adipose tissue browning, enhanced thermogenesis, and anti‐obesity effects in mice.

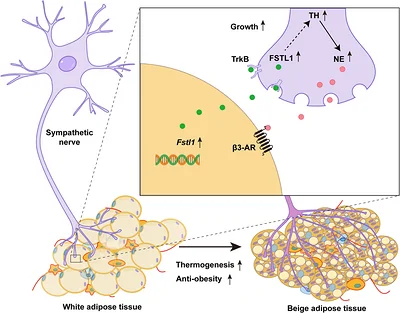

## 1 Introduction

The increasing prevalence of obesity has renewed interest in adipose tissue [1, 2]. Adipose tissue is recognized as a key metabolic and endocrine organ [3]. Brown and beige adipocytes are characterized by a multilocular morphology and numerous mitochondria containing uncoupling protein 1 (UCP1). They possess a high capacity for energy dissipation, predominantly through UCP1‐dependent and UCP1‐independent thermogenesis [4, 5, 6, 7, 8]. Thus, brown and beige adipocytes are defined as thermogenic adipocytes. Activation of thermogenic adipocytes represents a promising strategy for obesity treatment, owing to their ability to increase energy expenditure [9, 10, 11].

Thermogenic adipose tissues are richly innervated by sympathetic nerves. Sympathetic nerves activate thermogenic adipocytes through the release of norepinephrine (NE), which binds to β‐adrenergic receptors on adipocytes to activate protein kinase A and stimulate UCP1 activity [10]. In addition, sympathetic nerves secrete neuropeptide Y (NPY), which antagonizes NE function [12]. Conversely, thermogenic adipocytes secrete various factors that potentially regulate sympathetic innervation [13, 14]. Previous evidence indicates that adipocytes release several neurotrophic factors to promote sympathetic innervation, including nerve growth factor [15, 16], neuregulin 4 (NT4) [17], calsyntenin‐3β (Clstn3β) [18], and neurotrophic factor‐3 (NT3) [19], which promote sympathetic innervation. Additionally, adipocyte‐secreted bone morphogenetic protein 8b (BMP8b) induces browning and increases sympathetic innervation [20]. Adipokine leptin regulates sympathetic innervation of adipose tissue via the brain‐derived neurotrophic factor pathway [21]. Thermogenic adipocytes release zinc to promote sympathetic innervation and thermogenesis [22]. Furthermore, adipose‐resident immune cells secrete pro‐inflammatory and anti‐inflammatory cytokines that impact sensory and sympathetic nerves [10]. A recent study has demonstrated that anti‐inflammatory M2‐like macrophages secrete Slit3 to enhance sympathetic activity and thermogenesis in adipose tissue [23]. Therefore, elucidating the active adipokines secreted by adipocytes and exploring their roles in intercellular or inter‐organ crosstalk are essential for maintaining systemic metabolic homeostasis [24, 25].

Follistatin‐like 1 (FSTL1), also known as transforming growth factor‐β (TGF‐β)‐stimulated clone 36 (TSC‐36), was first identified in osteoblast cells in 1993 [26]. Previous studies have shown that FSTL1 modulates oviductal epithelial cell development [27], as well as lung development and alveolar maturation [28]. Elevated serum FSTL1 levels are correlated with disease activity in patients with rheumatoid arthritis, osteoarthritis, and cardiovascular diseases, suggesting FSTL1 as a useful serum biomarker for disease activity [29, 30, 31, 32, 33]. Huang et al. reported that deletion of the *Fstl1* gene or depletion of *Fstl1*‐expressing progenitors induces progenitor incompetence and interscapular brown adipose tissue (BAT) paucity in mice [34]. A recent study confirms that deleting FSTL1 specifically in agouti‐related peptide (AgRP) neurons of the hypothalamus increases food intake, reduces energy expenditure, and impairs insulin sensitivity in obese mice [35]. Conversely, overexpressing FSTL1 in AgRP neurons ameliorates the metabolic disorders in obese mice. These findings highlight a key role of FSTL1 as a central insulin sensitizer [35].

FSTL1, as a secreted protein, has also been recognized as a newly discovered adipokine [24, 36, 37]. First, FSTL1 is highly expressed during the early stages of pre‐adipocyte differentiation, as well as in mouse embryonic fibroblasts and stromal vascular fraction cells [38, 39, 40]. An in vitro study has also demonstrated the secretion of FSTL1 by pre‐adipocytes [41]. Second, FSTL1 is expressed in both BAT and white adipose tissue (WAT) depots [39]. Our in vitro study suggested FSTL1 as a novel stimulator of β‐adrenergic signaling to upregulate UCP1 expression in brown adipocytes [39]. Deletion of Fstl1 in progenitor and mature adipocytes inhibits adipogenesis in vitro and protects mice from diet‐induced obesity in vivo [40]. However, the roles of FSTL1 in beige adipocyte recruitment and its functions remain unknown. Given evidence that FSTL1 haploinsufficiency leads to impaired BAT thermogenesis, we hypothesized that FSTL1 could promote WAT browning and thermogenesis. To test this hypothesis, we assessed the browning and metabolic effects of FSTL1 in mice. We also evaluated the anti‐obesity effect of FSTL1 in mice fed a high‐fat diet (HFD). Finally, the underlying mechanisms of FSTL1 in WAT browning were determined. We found that FSTL1 induces WAT browning by promoting sympathetic innervation in adipose tissue. Additionally, FSTL1 can be endocytosed by sympathetic cells primarily via tropomyosin‐related kinase B (TrkB). This study demonstrates that FSTL1 is an adipose‐derived ‘adipokine‐like’ neurotrophic factor.

## 2 Results

### 2.1 Expression of FSTL1 in WAT is Closely Related to the Browning Process

To confirm the relationship between FSTL1 expression and WAT browning, we subjected mice to cold exposure or CL316,243 (CL) injections for 1 week. Numerous UCP1‐positive adipocytes with multilocular morphology were distributed throughout inguinal WAT (iWAT) of mice following cold exposure or CL treatment (Figure S1A). Cold exposure or CL treatment increased UCP1 protein and mRNA levels in inguinal WAT (iWAT) (Figure S1B,C). Similarly, FSTL1 protein and mRNA levels were increased in iWAT of mice upon browning stimuli (Figure S1B,C). Furthermore, we found that the density of FSTL1‐immunoreactive cells was simultaneously increased in iWAT of mice with cold exposure or CL treatment (Figure S1D), and FSTL1‐positive cells exhibited a multilocular morphology, similar to that of UCP1‐positive adipocytes (Figure S1E). These data indicate that increased FSTL1 expression is strongly associated with the browning process in iWAT.

### 2.2 FSTL1 Overexpression Promotes Browning in WAT and Enhances Energy Metabolism In Vivo

**FIGURE 1:**
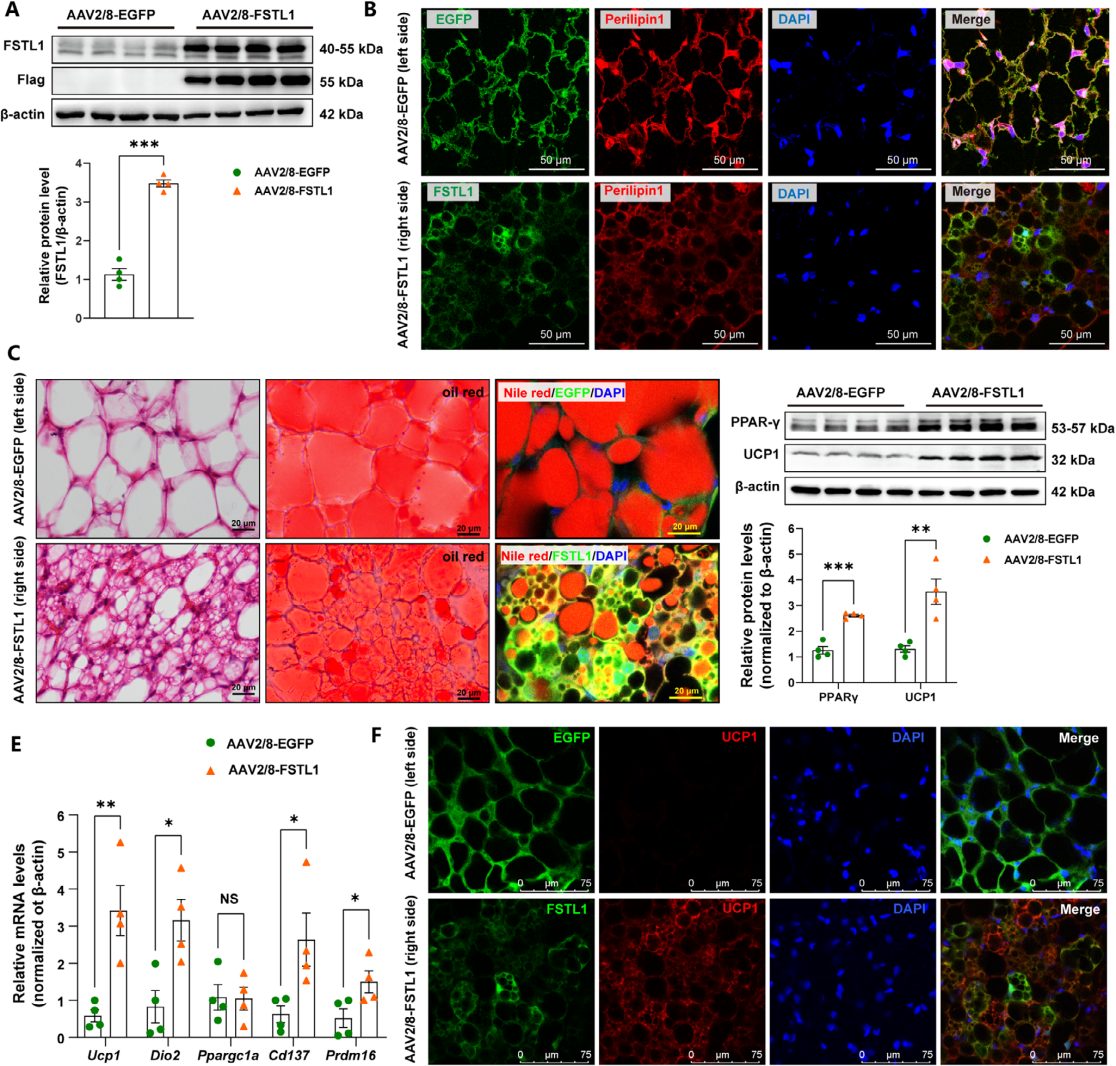
FSTL1 overexpression induces beige fat biogenesis in iWAT. (A) Immunoblot and quantification of FSTL1 and Flag proteins in iWAT of wild‐type mice with local delivery of AAV2/8‐FSTL1 (right side) and AAV2/8‐EGFP (left side) vectors at room temperature (*n* = 4 per group). (B, C) Representative images showing FSTL1 immunofluorescence staining (B), HE sections (C, left panel), Oil red O staining (C, middle panel), and Nile red staining (C, right panel) of iWAT as in (A) (*n* = 4 per group). (D) Immunoblot and quantification of PPARγ and UCP1 proteins in iWAT as in (A) (*n* = 4 per group). (E) Relative mRNA levels of indicated genes in iWAT as in (A) (n = 4 per group). (F) Representative images showing FSTL1 and UCP1 immunofluorescence staining of iWAT as in (A) (*n* = 4 per group). Data are presented as mean ± SEM. An unpaired Student's *t*‐test was used to make group comparisons. ^*^ *p* < 0.05, ^**^ *p* < 0.01, ^***^ *p* < 0.001 vs. AAV2/8‐EGFP side.

To directly validate the role of FSTL1 in WAT browning, Adeno‐associated virus 2/8 (AAV2/8)‐vectors carrying FSTL1 genes were locally injected into the right side of iWAT, and AAV2/8‐EGFP vectors were injected into the left side of iWAT as a self‐control. After 3 weeks of viral injection, the overexpression of FSTL1 in iWAT with AAV2/8‐FSTL1 injections was validated by western blotting (Figure 1A). Almost all FSTL1‐ or EGFP‐positive cells in iWAT were co‐labeled with perilipin 1, a marker of adipocytes (Figure 1B). AAV2/8‐FSTL1‐injected iWAT exhibited multilocular adipocytes containing numerous smaller lipid droplets (Figure 1C, lower panel). In addition, these multilocular adipocytes were co‐labeled with FSTL1 (Figure 1C, lower panel). Both UCP1 and peroxisome proliferator‐activated receptor γ (PPARγ) protein levels were increased in AAV2/8‐FSTL1‐injected iWAT (Figure 1D). The browning‐related genes were also distinguishable increased in AAV2/8‐FSTL1‐injected iWAT (Figure 1E). Furthermore, FSTL1‐positive cells with smaller lipid droplets were co‐labeled with UCP1 and exhibited strong fluorescent intensity of UCP1 in iWAT (Figure 1F). These data indicate that FSTL1 functions as a novel stimulator of WAT browning at room temperature.

**FIGURE 2:**
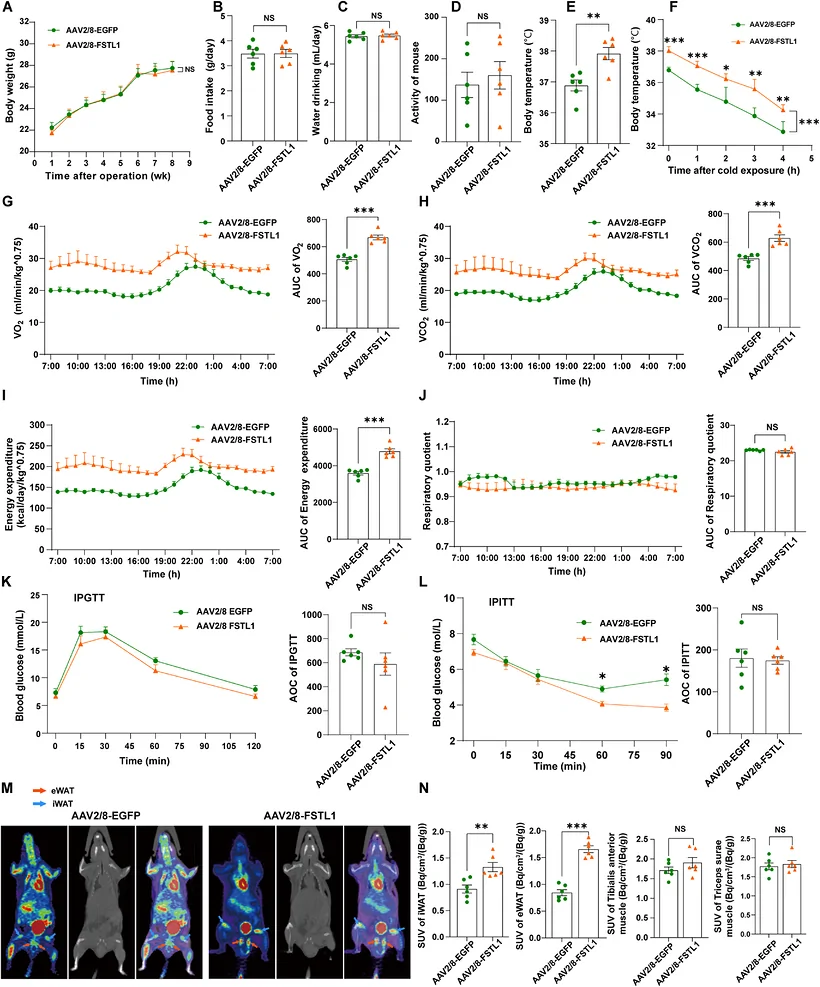
FSTL1 overexpression enhances energy metabolism in vivo. (A–E) The diagrams showing the body weight (A), food intake (B), water drinking (C), activity (D), and rectal temperature (E) of wild mice with local delivery of AAV2/8‐FSTL1 or AAV2/8‐EGFP vectors under room temperature (n = 6 per group). (F) The diagram showing the rectal temperature of mice as in (A) under cold exposure at indicated time points (*n* = 6 per group). (G–L) The diagrams showing the oxygen consumption (G), carbon dioxide production (H), energy expenditure (I), respiratory quotient (J), blood glucose at indicated time points after glucose (K) and insulin (L) injections of mice as in (A) (*n* = 6 per group). (M, N) Representative PET‐CT images and quantification (N) of 18F‐FDG in iWAT (blue arrows), eWAT (red arrows), tibials anterior, and triceps surae muscle of mice as in (A) (*n* = 6 per group). Data are presented as mean ± SEM. A two‐way repeated‐measures ANOVA was performed to make group comparisons in (A, F–L). An unpaired Student's *t*‐test was used to make group comparisons in (B–E, N). NS, not significant. ^**^ *p* < 0.01, ^***^ *p* < 0.001 vs. AAV2/8‐EGFP group.

To further investigate the physiological significance of FSTL1 on energy homeostasis, we bilaterally injected AAV2/8‐FSTL1 vectors into iWAT and epididymal WAT (eWAT) of mice and housed them at room temperature. Compared to the control mice injected with AAV2/8‐EGFP vectors, the AAV2/8‐FSTL1‐injected mice showed no significant difference in body weight, food intake, water drinking, and activity (Figure 2A–D). However, rectal temperature was significantly elevated in the AAV2/8‐FSTL1 injected mice (Figure 2E). Upon acute cold exposure, the AAV2/8‐FSTL1‐injected mice maintained significantly higher rectal temperatures than control mice, indicating enhanced cold tolerance (Figure 2F). Subsequently, we observed that the AAV2/8‐FSTL1‐injected mice exhibited increased oxygen consumption, carbon dioxide production, and energy expenditure, while respiratory quotient remained unchanged (Figure 2G–J). While fasting blood glucose levels and area of curve (AOC) showed no significant difference between groups (Figure 2K,L). To assess local glucose uptake in WAT, we performed 18F‐FDG PET‐CT quantitation. Imaging revealed significantly enhanced 18F‐FDG uptake specifically in iWAT and eWAT of the AAV2/8‐FSTL1‐injected mice (Figure 2M,N). In contrast, 18F‐FDG uptake in the tibialis anterior muscle and triceps surae muscle showed no significant differences between groups. Collectively, these findings demonstrate that adipose‐specific overexpression of FSTL1 enhances systemic energy expenditure and promotes regional glucose uptake specifically within WAT depots in vivo.

### 2.3 FSTL1 Overexpression Prevents Metabolic Dysfunction in Mice With Diet‐Induced Obesity

**FIGURE 3:**
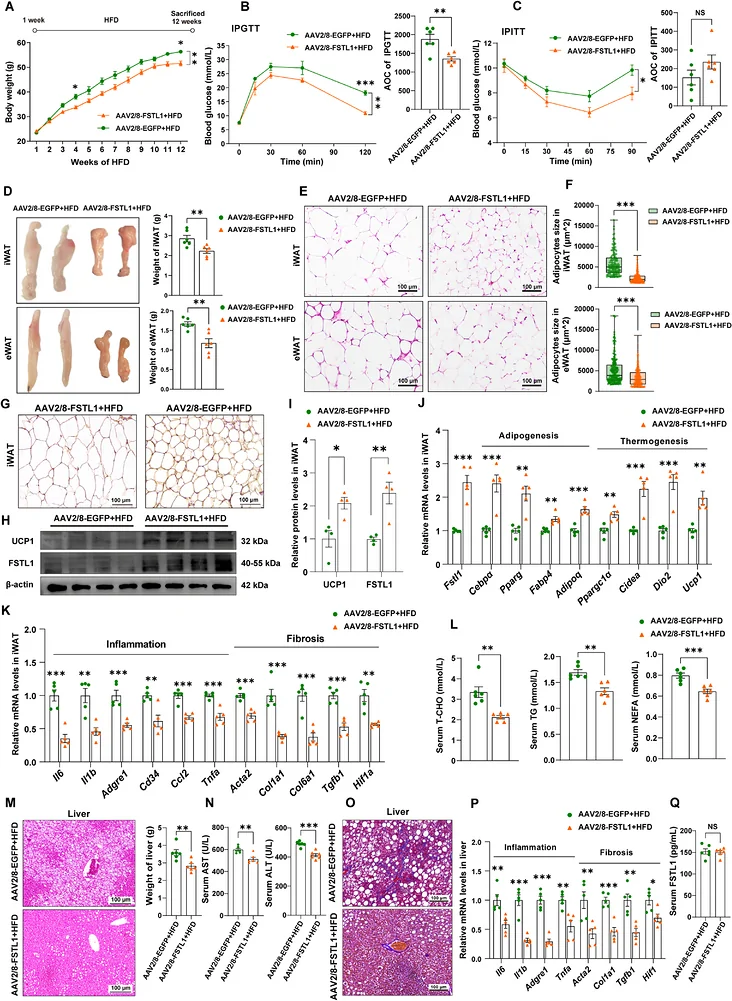
FSTL1 overexpression improves metabolic homeostasis in mice with diet‐induced obesity. (A) The diagram showing the body weight of wild mice with local delivery of AAV2/8‐FSTL1 or AAV2/8‐EGFP vectors under HFD conditions (*n* = 6 per group). (B, C) The diagrams showing blood glucose at indicated time points after glucose (B) and insulin (C) injections of mice shown in (A) (*n* = 6) per group. (D) Representative macroscopy images and weight quantification of iWAT and eWAT from mice in (A) (*n* = 6 per group. (E, F) Representative HE staining and the diagrams showing the adipocyte size of iWAT and eWAT from mice as in (A). (G–I) Representative images of UCP1 immunostaining (G) in iWAT as in (A) (*n* = 6 per group). (H, I) Immunoblot and quantification of UCP1 and FSTL1 in iWAT as in (A) (*n* = 4 per group). (J, K) Relative mRNA levels of adipogenesis, thermogenesis (J), inflammation, and fibrosis genes (K) in iWAT from mice as in (A) (*n* = 5 per group). (L) The diagram showing the concentration of total cholesterol (T‐CHO), triglycerides (TG), and non‐esterified free fatty acids (NEFA) in serum of mice shown in (A) (*n* = 6 per group). (M) Representative HE staining images and weight quantification of liver from mice as in (A) (*n* = 6 per group). (N) The diagrams showing the concentration of AST and ALT in serum of mice as in (A) (*n* = 6 per group). (O, P) Representative Masson staining images of the liver (O) and relative mRNA levels of inflammation and fibrosis genes in the liver (P) of mice as in (A) (*n* = 5 per group). (Q) The diagram showing FSTL1 protein levels in serum of mice from the AAV2/8‐EGFP+HFD and AAV2/8‐FSTL1+HFD groups (*n* = 6 per group). Data are presented as mean ± SEM. A two‐way repeated‐measures ANOVA was performed to make group comparisons in (A)‐(C). An unpaired Student's *t*‐test was used to make group comparisons for (D, F, I–L, N, P, Q). ^*^ *p* < 0.05, ^**^ *p* < 0.01, ^***^ *p* < 0.001 vs. AAV2/8‐EGFP+HFD group.

Based on the role of FSTL1 in WAT browning, we further investigated the functions of FSTL1 in the context of diet‐induced obesity. 8‐week‐old mice were injected with AAV2/8‐FSTL1 or AAV2/8‐EGFP vectors into iWAT and fed a high‐fat diet (HFD) for 12 weeks. Compared to the AAV2/8‐EGFP+HFD group, mice in the AAV2/8‐FSTL1+HFD group showed a significant reduction in HFD‐induced body weight gain, improved glucose tolerance, and enhanced insulin sensitivity (Figure 3A,C). Mice in the AAV2/8‐FSTL1+HFD group exhibited a significant decrease in fat mass gain and adipocyte size in iWAT and eWAT (Figure 3D–F). iWAT of the AAV2/8‐FSTL1+HFD mice showed increases in numbers of UCP1‐positive cells, UCP1 protein levels, mRNA levels of adipogenic and thermogenic genes, together with decreased levels of inflammation and fibrosis markers (Figure 3G–K). Moreover, mice in the AAV2/8‐FSTL1+HFD group had reduced serum levels of total cholesterol (T‐CHO), triglycerides (TG), and non‐esterified free fatty acids (NEFA) (Figure 3L). Notably, mice in the AAV2/8‐FSTL1+HFD group exhibited decreases in weight of liver, lipid accumulation, serum aspartate aminotransferase (AST) and alanine aminotransferase (ALT) levels, collagen deposition, and mRNA levels of inflammation and fibrosis markers, when compared with those in the AAV2/8‐EGFP+HFD group (Figure 3M–P). Serum FSTL1 protein levels did not differ between groups (Figure 3Q), indicating that FSTL1 overexpression in our model is primarily restricted to adipose tissue. Taken together, these data demonstrate that adipose‐specific overexpression of FSTL1 counteracts obesity‐induced metabolic dysfunction, reduces inflammation and fibrosis in adipose tissue and liver, and promotes WAT browning under obesogenic conditions.

To determine the therapeutic function of FSTL1 in HFD diet‐induced obese mice, 8‐week‐old mice were fed an HFD for 12 weeks and locally injected with AAV2/8‐FSTL1 vectors into iWAT. Similarly, mice in the HFD+AAV2/8‐FSTL1 group exhibited reduced HFD‐induced body weight gain, along with improved glucose tolerance and insulin sensitivity (Figure S2A–C). Although iWAT or eWAT mass did not differ significantly between groups (Figure S2D), adipocyte size was markedly reduced in both iWAT and eWAT of mice in the HFD+AAV2/8‐FSTL1 group (Figure S2E,F). Moreover, iWAT from the HFD+AAV2/8‐FSTL1 group displayed increased numbers of UCP1‐positive cells, elevated UCP1 protein levels (Figure S2G–I), enhanced mRNA expression of adipogenic and thermogenic genes (Figure S2J), and significantly lower expression of inflammatory and fibrosis markers (Figure S2K). Concomitantly, serum T‐CHO, TG, and NEFA were significantly reduced in the HFD+AAV2/8‐FSTL1 group (Figure S2L). Furthermore, these mice exhibited decreased hepatic lipid deposition, liver weight, serum AST and ALT levels, collagen deposition, and hepatic mRNA levels of inflammatory and fibrosis genes compared with the HFD+AAV2/8‐EGFP controls (Figure S2M–P). There was no difference in FSTL1 protein levels in serum between groups (Figure S2Q). These data indicate the therapeutic function of FSTL1 in diet‐induced obese mice.

### 2.4 Mice With Haploinsufficiency of FSTL1 in Preadipocytes Show Impaired Cold‐Induced Thermogenesis and are Prone to Obesity

**FIGURE 4:**
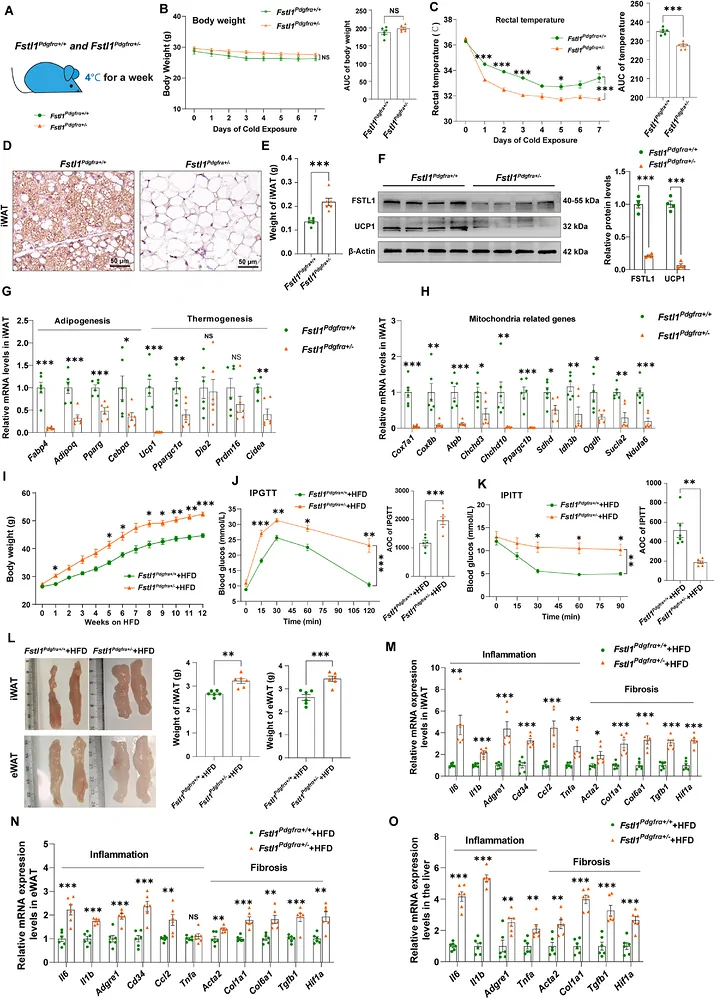
Mice with haploinsufficiency of FSTL1 in preadipocytes show impaired cold‐induced thermogenesis and are prone to obesity. (A) The image showing the experimental approach of mice. (B, C) The diagram showing the body weight (B) and rectal temperature (C) of mice under cold exposure (n = 6 per group). (D, E) Representative UCP1 immunostaining images (D) and weight quantification (E) of iWAT from mice as in (A) (*n* = 6 per group). (F) Representative immunoblot and quantification of FSTL1 and UCP1 protein in iWAT of mice as in (A) (*n* = 4 per group). (G, H) Relative mRNA levels of adipogenesis, thermogenesis (G), and mitochondria‐related genes (H) in iWAT from mice as in (A) (*n* = 6 per group). (I) The diagram showing the body weight of mice with HFD feeding for 12 weeks (*n* = 6 per group). (J, K) The diagrams showing blood glucose at indicated time points after glucose (J) and insulin (K) injections of mice as in (I) (*n* = 6 per group). (L) Representative macroscopy images and weight quantification of iWAT and eWAT from mice as in (I) (*n* = 6 per group). (M, N) Relative mRNA levels of inflammation and fibrosis genes in iWAT, eWAT, and liver of mice as in (I) (*n* = 6 per group). (O) Relative mRNA levels of inflammation and fibrosis genes in the liver of mice as in (I) (*n* = 6 per group). Data are presented as mean ± SEM. A two‐way repeated‐measures ANOVA was performed to make group comparisons in (B, C, I–K). An unpaired Student's *t*‐test was used to make group comparisons (E–H, L–O). ^*^ *p* < 0.05, ^**^ *p* < 0.01, ^***^ *p* < 0.001 vs. *Fstl1^Pdgfrα+/+^* group.

As the above data highlight that FSTL1 is an important regulator of WAT browning, we then assessed whether FSTL1 deficiency affects WAT browning. To this end, we generated mice in which FSTL1 was deleted in platelet‐derived growth factor receptor α (PDGFRα^+^) cells (Figure S3A). As the homozygous pups (*Fstl1^Pdgfrα−/−^*) showed a skeletal defect after birth, the heterozygous mice (*Fstl1^Pdgfrα^* ^+/−^) without any obvious physiological abnormalities were used in this study. To determine the physiological relevance of FSTL1, we then exposed the heterozygous mice to cold conditions for 1 week (Figure 4A). Although no significant difference was observed in body weight between groups (Figure 4B), *Fstl1^Pdgfrα^* ^+/−^ mice showed a rapid decrease in rectal temperature (Figure 4C). Accordingly, lots of UCP1‐positive adipocytes were observed in the iWAT and eWAT of *Fstl1^Pdgfrα^* ^+/+^ mice, but not *Fstl1^Pdgfrα^* ^+/−^ mice (Figure 4D and Figure S3B). *Fstl1^Pdgfrα^* ^+/−^ mice showed an increase in the weight of iWAT and eWAT (Figure 4E and Figure S3C). The iWAT and eWAT of *Fstl1^Pdgfrα^* ^+/−^ mice showed a significant decrease in UCP1 protein levels, together with decreased levels of adipogenic, thermogenic, and mitochondrial‐related genes (Figure 4F–H and Figure S3D–F). Interestingly, BAT of *Fstl1^Pdgfrα^* ^+/−^ mice showed a scattered distribution of UCP1‐positive adipocytes, a decrease in UCP1 protein levels, as well as reduced mRNA levels of adipogenic, thermogenic, and mitochondrial‐related genes (Figure S3G–K). Moreover, *Fstl1^Pdgfrα^* ^+/−^ mice fed an HFD gained more body weight than *Fstl1^Pdgfrα^* ^+/+^ mice (Figure 4I). Importantly, *Fstl1^Pdgfrα^* ^+/−^ mice displayed decreased glucose tolerance and insulin sensitivity, with increased iWAT and eWAT mass, increased adipocyte size, as well as increased mRNA levels of inflammation and fibrosis genes in iWAT and eWAT (Figure 4J–N and Figure S3L). The liver of *Fstl1^Pdgfrα^* ^+/−^ mice exhibited more lipid, as well as increased mRNA levels of inflammation and fibrosis genes (Figure S3L and Figure 4O). Taken together, the above data indicate that the loss of FSTL1 specifically in PDGFRα^+^ cells reduces the capacity of WAT browning and worsens obesity.

### 2.5 FSTL1 Cannot Induce Adipocytes Browning Directly In Vitro

In light of the upregulation of PPARγ in AAV2/8‐FSTL1‐injected iWAT (Figure 1D), we hypothesized that FSTL1 overexpression can promote differentiation of WAT‐derived precursor cells into UCP1^+^ adipocytes. To confirm this hypothesis, we investigated the roles of rFSTL1 in adipose‐derived stem Cells (ADSCs) and 3T3‐L1 in vitro. Unexpectedly, there was no difference in UCP1 protein expression and mRNA levels of browning‐related genes in ADSCs from iWAT exposed to rFSTL1 treatment when compared with the control group (Figure S4A,B). However, significantly increased levels of UCP1 protein and mRNA, and browning‐related gene mRNA levels were present in ADSCs from iWAT exposed to Rosiglitazone when compared with other groups (Figure S4A,B). Similar tendencies were present in ADSCs from eWAT and 3T3‐L1 exposed to rFSTL1 treatment when compared with control or Rosiglitazone groups (Figure S4C–F). These data demonstrate that rFSTL1 cannot promote the browning process in vitro. We next investigated whether FSTL1 overexpression can promote browning in vitro. An elevation of UCP1 protein and browning‐related marker levels in ADSCs from iWAT was only present in the Rosiglitazone group, but not the lenti‐FSTL1 group, when compared with the control group (Figure S4G,H). Consistent with the above results, similar tendencies of UCP1 protein and browning‐related gene mRNA levels were detected in ADSCs from eWAT and 3T3‐L1 (Figure S4I–L). Collectively, these data provide further confirmation that FSTL1 cannot directly promote the browning process in vitro.

### 2.6 FSTL1 Overexpression Regulates Sympathetic Innervation in WAT

**FIGURE 5:**
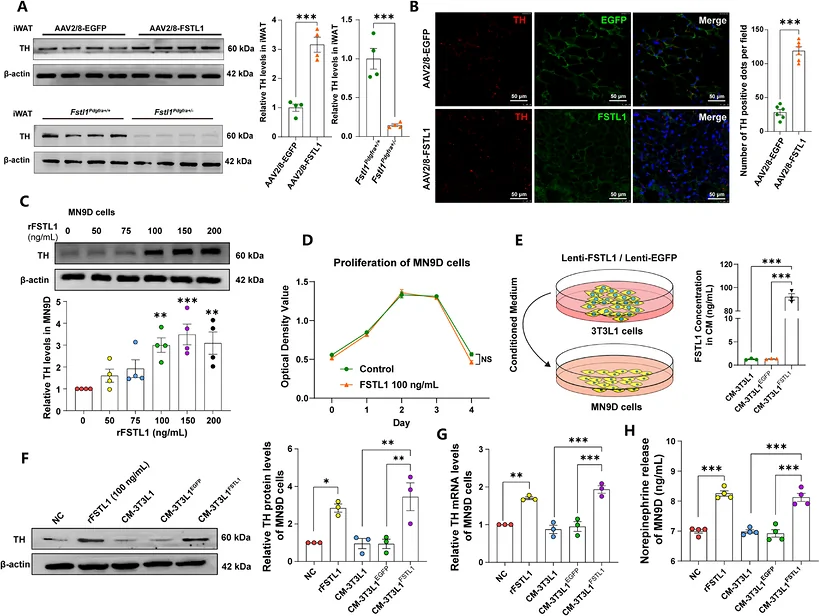
FSTL1 regulates the growth and innervation of sympathetic neurons in vivo. (A) Immunoblot and quantification of TH proteins in iWAT of wild‐type mice with locally injected AAV2/8‐FSTL1 (right side) and AAV2/8‐EGFP (left side) vectors (upper panel) or *Fstl1^Pdgfra+/+^* and *Fstl1^Pdgfra±^* mice (lower panel) at room temperature (*n* = 4 per group). (B) Representative images showing TH immunofluorescence staining and quantification of TH‐positive fibers in iWAT of AAV2/8‐FSTL1 (right side) and AAV2/8‐EGFP (left side) injections (n = 6 per group). Scale bar = 50 µm. (C) Immunoblot and quantification of TH protein in MN9D cell lines treated with rFSTL1 at indicated concentrations (*n* = 4 per group). (D) Diagram showing the proliferation curve of MN9D cells treated with rFSTL1 at 100 ng/mL (*n* = 4 per group). (E) Image showing the experimental approach to determine the effects of FSTL1 released by FSTL1‐overexpressing adipocytes on MN9D cell lines and diagram showing the concentration of FSTL1 in conditioned medium (CM) from 3T3L1 cell lines with FSTL1 overexpression (*n* = 3 per group). (F) Immunoblot and quantification of TH protein in MN9D cells treated with rFSTL1 or CM from 3T3‐L1 cells (*n* = 3 per group). (G) Relative mRNA levels of TH in MN9D cell lines as in (F) (*n* = 3 per group). (H) Diagram showing the concentration of norepinephrine released from MN9D cells in indicated groups (*n* = 4 per group). Data are presented as mean ± SEM. An unpaired two‐tailed Student's *t*‐ test was used for group comparisons in (A, B). NS, not significant. ^*^ *p* < 0.05, ^**^ *p* < 0.01, ^***^ *p* < 0.001 vs. the AAV2/8‐EGFP group or the control group. A one‐way ANOVA followed by Tukey's test was used for group comparisons in (C, E–H). A two‐way repeated‐measures ANOVA was used for group comparison in (D). ^*^ *p* < 0.05, ^**^ *p* < 0.01, ^***^ *p* < 0.001 vs. the indicated group or the control group.

Given that FSTL1 overexpression induces browning of WAT in vivo, but not in vitro, we hypothesized that FSTL1 may indirectly induce browning through other cells in vivo. Sympathetic nerves are critical in the activation of thermogenic adipocytes [42, 43, 44, 45, 46]. Therefore, we aimed to determine whether FSTL1 promotes sympathetic innervation in WAT. We found an increase in Tyrosine Hydroxylase (TH) protein levels in iWAT of the AAV2/8‐FSTL1 injected mice when compared with AAV2/8‐EGFP injected mice (Figure 5A). Similarly, FSTL1 overexpression also led to an increase in TH protein levels in iWAT of mice with diet‐induced obesity (Figure S5A–D). Mice with haploinsufficiency of FSTL1 in preadipocytes show decreased TH protein levels in iWAT (Figure 5A). In addition, we evaluated TH fiber distribution in iWAT. We found that the number of TH‐positive fibers was greater in iWAT of the AAV2/8‐FSTL1‐injected group (Figure 5B). We then further studied the effects of FSTL1 on sympathetic cells in vitro. We found that rFSTL1 (75, 100, 150, and 200 ng/mL) treatments increased TH protein expression levels in mouse dopaminergic cells (MN9D cells) (Figure 5C). However, the proliferation was not changed in MN9D cells with rFSTL1 treatments at 100 ng/mL (Figure 5D). To confirm the effects of FSTL1 released by FSTL1‐overexpressing adipocytes on sympathetic cells, the conditioned medium (CM) of FSTL1‐overexpressing 3T3L1 cells (CM‐3T3L1^FSTL1^) was collected, concentrated, and added to the medium of MN9D cells (Figure 5E). An elevation of FSTL1 concentrations in CM from CM‐3T3L1^FSTL1^ cells was validated by ELISA (Figure 5E). We observed significant increases of TH protein and mRNA expression levels in MN9D cells of the CM‐3T3L1^FSTL1^ and rFSTL1 groups, when compared with the other three groups (Figure 5F,G). TH protein and mRNA expression levels were comparable in MN9D cells between the CM‐3T3L1^FSTL1^ and rFSTL1 groups (Figure 5F,G). We also found that the levels of NE synthesized and released by sympathetic neurons were significantly increased in the medium of MN9D cells in the CM‐3T3L1^FSTL1^ and rFSTL1 groups, when compared with the other groups (Figure 5H). The levels of NE released were comparable in the medium of MN9D cells between the CM‐3T3L1^FSTL1^ and rFSTL1 groups (Figure 5H). Collectively, these data demonstrate that FSTL1 can promote sympathetic innervation in vivo and in vitro.

**FIGURE 6:**
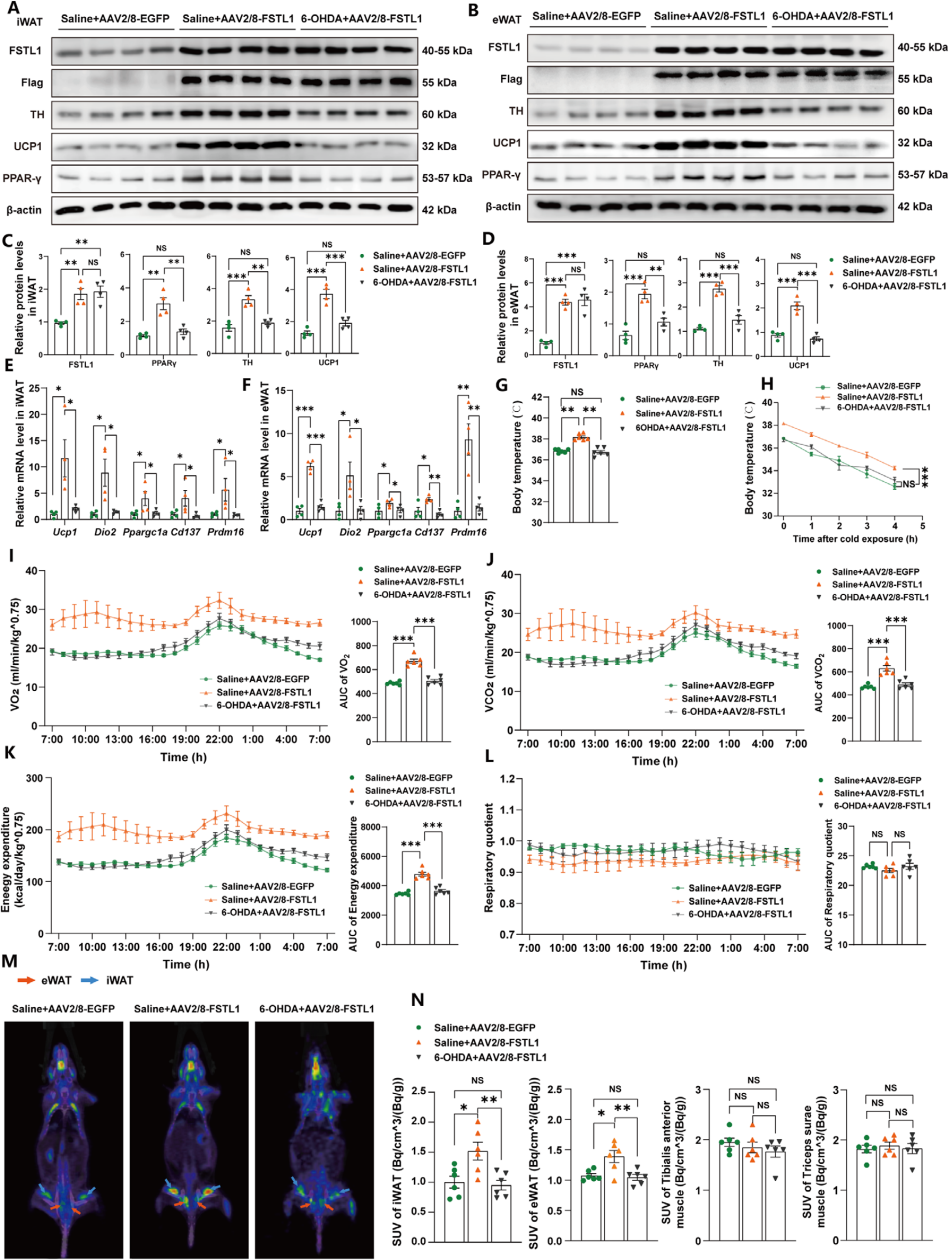
Sympathetic nerves denervation in adipose tissue blocks FSTL1‐induced browning. (A–D) Immunoblot and quantification of FSTL1, Flag, TH, UCP1, and PPAR‐γ proteins in iWAT (A, C) and eWAT (B, D) of wild‐type mice with 6‐OHDA or saline treatments, followed by local delivery of AAV2/8‐FSTL1 or AAV2/8‐EGFP under room temperature (*n* = 4 per group). (E, F) The diagrams showing browning‐related genes in iWAT (E) and eWAT (F) of mice as in (A) and (B) (*n* = 4 per group). (G) The diagram showing rectal temperature of mice as in (A) under room temperature (*n* = 6 per group). (H) The diagram showing rectal temperature of mice as in (A) under cold exposure at indicated time points (*n* = 6 per group). (I–L) The diagrams showing oxygen consumption (I), carbon dioxide production (J), energy expenditure (K), and respiratory quotient (L) of mice as in (A) (*n* = 6 per group). (M, N) Representative PET‐CT images and quantification of 18F‐FDG in iWAT (blue arrows), eWAT (red arrows), tibials anterior muscle, and triceps surae muscle of mice as in (A) (*n* = 6 per group). Data are presented as mean ± SEM. A one‐way ANOVA followed by a Tukey's test was used to make group comparisons in (A–G). A two‐way repeated‐measures ANOVA was used for group comparison in (H–L). NS, not significant. ^*^ *p* < 0.05, ^**^ *p* < 0.01, ^***^ *p* < 0.001 vs. indicated groups.

To further confirm that FSTL1 induces WAT browning via promoting sympathetic innervation, we performed sympathetic denervation of WAT by 6‐hydroxydopamine (6‐OHDA, a neurotoxin to selectively destroy sympathetic nerves) locally injections. TH protein levels were dramatically decreased in the iWAT and eWAT after 6‐OHDA injections, indicating the success of sympathetic denervation (Figure 6A,B). Both FSTL1 and Flag protein levels in iWAT were increased in the saline+AAV2/8‐FSTL1 and 6‐OHDA+AAV2/8‐FSTL1 groups. UCP1 protein and PPARγ protein levels were decreased in the 6‐OHDA+AAV2/8‐FSTL1 group when compared with the saline+AAV2/8‐FSTL1 group (Figure 6A–D). The expression of browning‐related genes was also decreased in the 6‐OHDA+AAV2/8‐FSTL1 group when compared with the saline+AAV2/8‐FSTL1 group (Figure 6E,F). The body weight, food intake, water drinking, and locomotor activity were not changed among groups (Figure S6A–D). However, rectal temperature was decreased in the 6‐OHDA+AAV2/8‐FSTL1 group when compared with the Saline+AAV2/8‐FSTL1 group (Figure 6G). Rectal temperature on cold exposure was also rapidly reduced in the 6‐OHDA+AAV2/8‐FSTL1 group, when compared with the Saline+AAV2/8‐FSTL1 group (Figure 6H). The data of metabolic cages showed a decrease in oxygen consumption, carbon dioxide production, and energy expenditure, but not respiratory quotient, in the 6‐OHDA+AAV2/8‐FSTL1 group, when compared with the Saline+AAV2/8‐FSTL1 group (Figure 6I–L). 18F‐FDG uptake was reduced in iWAT and eWAT of mice in the 6‐OHDA+AAV2/8‐FSTL1 group, when compared with those in the Saline+AAV2/8‐FSTL1 group. However, there was no difference in 18F‐FDG uptake of tibialis anterior and triceps surae muscles among groups (Figure 6M,N). Collectively, these results further demonstrate that adipose‐specific expression of FSTL1 induces the browning process, enhances energy metabolism and regional glucose uptake of WAT via promoting sympathetic innervation.

### 2.7 TrkB is the Main Receptor for Sympathetic Cells to Endocytose FSTL1

**FIGURE 7:**
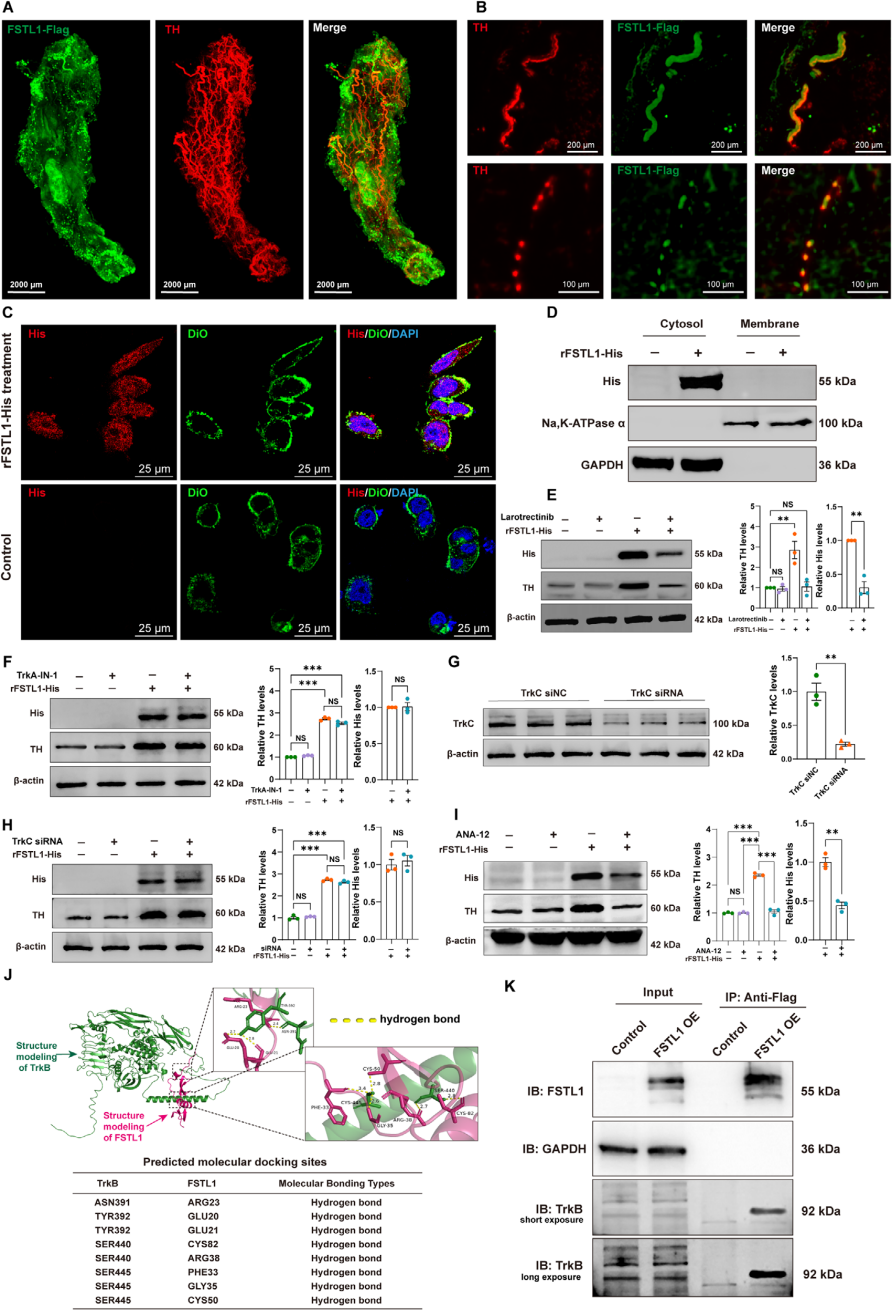
TrkB is the main receptor for sympathetic cells to take up FSTL1 in vitro. (A) Representative three‐dimensional images showing TH (red) and Flag (green) staining in iWAT of mice with AAV2/8‐FSTL1 local delivery. Scale bar = 2000 µm for (A); 200 µm for the upper panel in (B); 100 µm for the lower panel in (B). (B) Representative images showing the colocalization of TH‐positive fibers (red) and Flag protein (green). (C) Immunofluorescence images showing rFSTL‐His staining in MN9D cells exposed to rFSTL‐His treatments. (*n* = 3 per group). Scale bar = 25 µm. (D) Immunoblot analysis of rFSTL‐His protein in the cytosol of MN9D cells with rFSTL1‐His treatment (*n* = 3 per group). (E) Immunoblot and quantitative assessment of rFSTL‐His and TH proteins in MN9D cells subjected to rFSTL1 treatment (with or without) and pan‐Trk inhibitor larotrectinib (with or without) (*n* = 3 per group). (F) Immunoblot and quantitative evaluation of His and TH proteins in MN9D cells treated with rFSTL1 (with or without) and TrkA‐IN‐1 (with or without) (*n* = 3 per group). (G) Immunoblot and quantitative analysis of TrkC in MN9D cells to evaluate the efficiency of TrkC siRNA (*n* = 3 per group). (H) Immunoblot and quantitative assessment of rFSTL1‐His and TH proteins in MN9D cells treated with rFSTL1 (with or without) and TrkC siRNA (with or without). (*n* = 3 per group) (I) Immunoblot and quantitative evaluation of rFSTL1‐His and TH proteins in MN9D cells subjected to rFSTL1 treatment (with or without) and ANA12 (with or without) (*n* = 3 per group). (J) The docking diagram derived from structural modeling illustrates the binding region between TrkB (green) and FSTL1 (brown). (K) CO‐IP assessment showing interaction between FSTL1 and TrkB in 293T cells. Data are presented as mean ± SEM. An unpaired two‐tailed Student's *t*‐test was used for group comparisons in (G). NS, not significant. ^*^ *p* < 0.05, ^**^ *p* < 0.01, ^***^ *p* < 0.001 vs. the indicated group. A one‐way ANOVA followed by Tukey's test was used for group comparisons in (E, F, H, I). ^*^ *p* < 0.05, ^**^ *p* < 0.01, ^***^ *p* < 0.001 vs. the indicated group.

Our above results demonstrated that FSTL1 promotes sympathetic nerve innervation in vivo and in vitro. Therefore, we hypothesized that FSTL1 is an adipose tissue‐derived ‘neurotrophic factor’ adipokine. In conventional neurotrophic factor studies, these factors can be retrogradely transported along the axon. Thus, we immunostained sympathetic nerves in adipose tissue with antibodies against TH and Flag using a whole‐mount clearing approach in AAV2/8‐FSTL1 mice (Figure 7A). The colocalization of TH‐positive fibers and Flag was present in iWAT of mice from the AAV2/8‐FSTL1 group, indicating the endocytosis and retrograde trafficking processes of FSTL1 (Figure 7B). Of note, iWAT of mice in the AAV2/8‐FSTL1 group exhibited more dense TH‐positive fibers than those in the AAV2/8‐EGFP group (Video S1). To further confirm the endocytosis process of FSTL1, MN9D cells were treated with rFSTL1‐His protein in vitro. We found that rFSTL1‐His protein was present in the cytoplasm of MN9D cells (Figure 7C,D). As tropomyosin receptor kinase (Trk) recepters, includingTrkA, TrkB, and TrkC located on axons are fundamental for endocytic trafficking of neurotrophic factors, we then further investigated the receptors of FSTL1 on axons responsible for its endocytic trafficking. Western blot analysis showed that the levels of endocytic FSTL1 and TH protein were reduced in MN9D cells with Larotrectinib (a pan‐Trk inhibitor) and rFSTL1‐His treatment (Figure 7E). However, both endocytic FSTL1 and TH protein levels were not changed in MN9D cells with TrkA‐IN‐1 (a TrkA inhibitor) treatment (Figure 7F) and TrkC knockdown (Figure 7G,H). We found that endocytic FSTL1 and TH protein levels were reduced in MN9D cells with ANA12 (a TrkB inhibitor) and rFSTL1‐His treatment, when compared to rFSTL1‐His treatment alone (Figure 7I). We subsequently applied SWISS‐MODEL to predict the potential interaction domains between TrkB and FSTL1. The hydrogen bonds were predicted between TrkB and FSTL1 (Figure 7J). Co‐IP results showed the interaction between FSTL1 and TrkB in vitro (Figure 7K). Furthermore, colocalization of rFSTL1‐His and TrkB was observed in MN9D cells (Figure S7A). These data identify TrkB as the major contributor to endocytic trafficking of FSTL1 in sympathetic cells in vitro. We further investigated the canonical signaling pathways FSTL1‐activated TrkB. We found that phosphorylated ERK (p‐ERK) was significantly increased after FSTL1 treatment in vitro (Figure S7B). To further validate the distribution of recombinant FSTL1 after endocytosis, we performed cytoplasmic/nuclear fractionation followed by Western blot analysis. Notably, recombinant FSTL1 was found to localize in the nuclear fraction of MN9D cells, suggesting the potential nuclear functions beyond its canonical role as a secreted protein (Figure S7C). We further investigated whether FSTL1, as a neurotrophic factor, can regulate TrkB expression. We found that TrkB mRNA levels were significantly increased in MN9D cells after FSTL1 treatment (Figure S7D). While NT3, NT4, and BDNF (Brain‐Derived Neurotrophic Factor) protein levels maintain comparable in adipose tissue of mice with FSTL1 gene overexpression or knockdown, suggesting a major contribution of FTSL1 in enhancing sympathetic innervation (Figure S8).

### 2.8 TrkB is Required for FSTL1‐Induced Browning In Vivo

**FIGURE 8:**
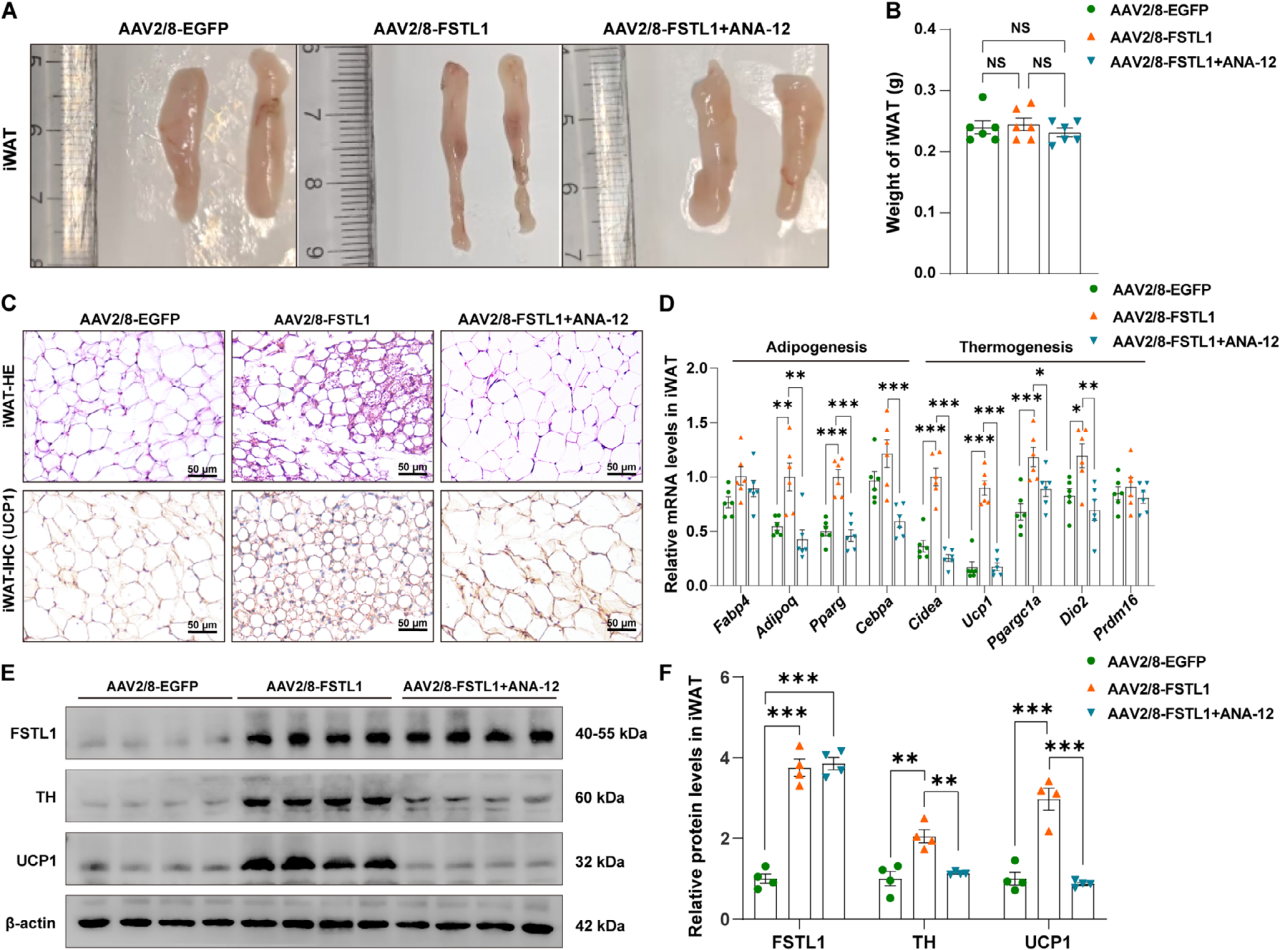
TrkB is required for FSTL1‐induced browning in vivo. (A) Representative images of iWAT from mice locally injected with either AAV2/8‐EGFP, AAV2/8‐FSTL1, or AAV2/8‐FSTL1 followed by systemic ANA‐12 peritoneal injections. (B) The diagram showing the weight of iWAT in mice as in (A) (*n* = 6 per group). (C) Representative images showing HE and UCP1 immunostaining in iWAT from mice as in (A). (D) The diagram showing the relative mRNA levels of adipogenesis and thermogenesis genes in iWAT from mice as in (A) (*n* = 6 per group). (E, F) Immunoblotting and quantitative analysis of FSTL1, TH, and UCP1 in iWAT from mice as in (A) (*n* = 4 per group). A one‐way ANOVA followed by a Tukey's test was used to make group comparisons. NS, not significant. ^*^ *p* < 0.05, ^**^ *p* < 0.01, ^***^ *p* < 0.001 vs. indicated group.

We next studied whether TrkB was required for FSTL1‐induced browning in vivo. Mice treated with AAV2/8‐FSTL1 vectors and ANA‐12 injections showed no differences in fat mass when compared with mice in other groups (Figure 8A,B). However, iWAT of mice in the AAV2/8‐FSTL1+ANA‐12 group showed a significant decrease in UCP1‐positive cells (Figure 8C), together with significantly decreased levels of adipogenic and thermogenic genes (Figure 8D), when compared with those in the AAV2/8‐FSTL1 group, but not the AAV2/8‐EGFP group. Mice in the AAV2/8‐FSTL1+ANA‐12 group also had significant decreases in UCP1 and TH protein levels, when compared with those in the AAV2/8‐FSTL1 group, but not the AAV2/8‐EGFP group (Figure 8E,F). These data indicate TrkB as the important receptor for FSTL1‐regulated browning and sympathetic innervation in vivo.

## 3 Discussion

Our study first reports that FSTL1 promotes WAT browning, enhances energy metabolism, and increases glucose uptake in male mice under room temperature. We confirm that increasing FSTL1 expression in iWAT promotes beige adipocyte biogenesis, improves metabolic homeostasis, and counteracts obesity. We further confirm that mice with haploinsufficiency of FSTL1 in preadipocytes are cold‐intolerant and prone to obesity. However, FSTL1 cannot induce adipocyte browning in vitro. Our data further show that FSTL1 can promote sympathetic innervation in vivo and in vitro. Additionally, deletion of sympathetic nerves blocks the function of FSTL1, including the browning process, energy metabolism, and regional glucose uptake. Our study further reveals that FSTL1 can be taken up by axons of sympathetic neurons primarily via TrkB. It is the first attempt to understand that FSTL1 induces WAT browning through promoting sympathetic innervation. The current data for the first time identify FSTL1 as an adipose‐derived ‘neurotrophic factor’ adipokine.

Several lines of evidence have shown that FSTL1 is a potential regulator of metabolism. Circulating FSTL1 levels exhibit dynamic regulation across different stages of obesity [38, 47]. As an important hallmark of adipogenic conversion, FSTL1 expression transiently surges in early‐stage preadipocytes and embryo fibroblast differentiation [38, 40, 41]. Furthermore, FSTL1 can directly promote brown adipocytes thermogenesis via activating β‐adrenergic signaling [39]. Moreover, FSTL1 directly promotes adipogenesis during WAT development through integrin/FAK/ERK signaling [40]. In BAT involution, loss of the FSTL1 gene or FSTL1‐expressing progenitors leads to brown adipogenic incompetence and BAT paucity in mice [34, 39]. In this study, we further expand the beneficial effect of FSTL1 in adipose tissues. We first determine that FSTL1 promotes WAT browning, energy expenditure, and regional glucose uptake, while its haploinsufficiency impairs cold‐induced browning. These lines of evidence indicate that FSTL1 has different modes of action in BAT (involution and thermogenesis) and WAT (adipogenesis and browning). Moreover, iWAT‐specific overexpression of FSTL1 ameliorates metabolic disorders in mice with diet‐induced obesity, suggesting its therapeutic potential for obesity.

Adipose tissues are innervated by sympathetic nerves, which is critical in mediating thermogenesis and lipolysis [42, 43, 44, 45, 46]. Increasing evidence reveals several factors from adipocytes that can promote the sympathetic innervation of adipose tissue [13, 14, 48], including BMP8b and nerve growth factor from brown/white adipocytes [15, 16, 20, 49, 50, 51], neuregulin 4 from white adipocytes [17], Clstn3β from brown adipocytes [18, 20] neurotrophic factor 3 from brown/beige adipocytes [19], BDNF from SVF of iWAT, Zinc from adipocytes [22], semaphoring 7A from adipocytes [52], Slit guidance ligand 3 from adipocyte progenitors [52], and Olfactomedin‐4 from brown adipocytes [53]. As other reports suggest, FSTL1 may play an important role during the development and disorders of the central nervous system [43, 54]. Therefore, we hypothesize that FSTL1, as a secreted protein, may induce WAT browning via promoting sympathetic nerve distribution. FSTL1 overexpression promotes dense distribution of sympathetic nerves in adipose tissue; haploinsufficiency of FSTL1 leads to decreased TH protein levels in adipose tissue. In addition, rFSTL1 protein increases TH expression in sympathetic cells in vitro. Moreover, FSTL1 secreted by adipocytes can increase TH expression and NE secretion in vitro. Local denervation using 6‐OHDA can block the beneficial effect of FSTL1 in male mice. These lines of evidence demonstrate that FSTL1 is a newly identified fat‐derived neurotrophic factor mediating adipocyte‐sympathetic nerve crosstalk. Previous studies support an important role of FSTL1 in the central nervous system [43, 55]. FSTL1 is strongly expressed in the developing telencephalon, diencephalon, brainstem, and spinal cord [54]. FSTL1 deletion leads to an abnormal distribution and migration of cortical upper‐layer neurons in the developing cerebral cortex [56]. In addition, FSTL1, secreted from nociceptive afferent terminals of small‐diameter dorsal root ganglion (DRG) neurons, inhibits excitatory synaptic transmission by activating Na^+^, K^+^‐ATPase, thereby gating the threshold of somatic sensation [57]. The current study provides a new insight into issues related to FSTL1 in the peripheral nervous system. To the best of our knowledge, this is the first study to demonstrate that adipocyte‐derived FSTL1 regulates sympathetic innervation of adipose tissue, revealing a previously unexplored player in adipose tissue innervation. Of note, a very recent study identified two distinct types of sympathetic neurons (parenchymal and vascular projections) innervating interscapular BAT [58], which has attracted considerable attention [59, 60]. In that study, Neri et al. demonstrated that parenchymal projections regulate interscapular BAT thermogenesis, whereas vascular projections regulate glucose uptake in interscapular BAT. Building on these findings, it would be of great interest to investigate whether the sympathetic innervation of beige fat is distinct as well, given that beige fat is also involved in thermogenesis and glucose uptake. Accordingly, it remains possible that FSTL1 also promotes distinct patterns of sympathetic innervation in interscapular BAT or beige fat.

Receptor‐medicated endocytosis and retrograde trafficking processes are fundamental features of neurotrophin signaling [61]. We have identified that FSTL1 can be taken up by sympathetic cells and nerve fibers. We then further identified the receptors medicating of FSTL1 endocytosis. Previous findings have identified DIP2A as a potential receptor in cardiomyocytes [62, 63] and tumor cells [64], TGF‐β1 signaling in lung development and pulmonary fibrosis [28, 65], and TLR4 in inflammatory response [66]. Recently, FSTL1 has been reported to reverse liver fibrosis via DIP2A/CD14 receptor [67]. Other receptors also exist to be related to FSTL1 [55, 67]. In this study, we first demonstrate that TrkB is the main receptor mediating endocytic trafficking of FSTL1 and is required for FSTL1‐induced sympathetic innervation and WAT browning. The canonical downstream signaling pathways of TrkB activated by neurotrophins include the PI3 kinase/protein kinase B (PI3K/Akt), mitogen‐activated protein kinase/extracellular signal‐regulated Kinase (MAPK/ERK), and protein kinase C (PKC) cascades [68]. FSTL1, as a newly identified neurotrophic factor, can primarily activate the MAPK/ERK pathway, as evidenced by the increased levels of phosphorylated ERK (p‐ERK) after FSTL1 treatments in vitro. Furthermore, FSTL1 is also detected in the nucleus of sympathetic cells, raising the possibility of potential nuclear functions beyond its canonical role as a secreted protein. However, it remains unclear whether the nuclear functions of FSTL1 are also involved in promoting sympathetic distribution in the browning process. Additionally, although FSTL1 overexpression or deficiency did not affect BDNF, NT4, and NT3 mRNA levels, we cannot exclude their contribution to sympathetic distribution in adipose tissue with FSTL1 overexpression. Notably, FSTL1 leads to elevated TrkB levels, which might amplify the biological actions of other ligands of TrkB even if they remain comparable.

Recently, a central study has shown that FSTL1 is a central insulin sensitizer, as evidenced by the findings that FSTL1 signaling in AgRP neurons regulates metabolic balance [35]. Our study further indicates that FSTL1 can exert effects on WAT browning in a paracrine manner. First, we used self‐controlled experiments to investigate the effect of FSTL1 in mice. Second, our in vitro data indicate that adipocytes can promote sympathetic nerve growth and NE release in a paracrine manner by glycoprotein FSTL1 secretion. Thirdly, food intake and circulating FSTL1 protein levels were not altered between groups, suggesting an insufficiency to act on the central control of feeding behavior. Therefore, FSTL1 can serve as a local mediator of intercellular communication between adipocytes and sympathetic nerves.

In addition to sympathetic innervation, the role of sensory innervation in adipose tissue is receiving increasing attention [69, 70, 71, 72, 73]. The study by Makwana et al. has demonstrated that α‐calcitonin gene‐related peptide sensory neurons, by inhibiting thermogenesis, are a potential target for obesity treatments [74]. Loss of adipose mammalian target of rapamycin complex 2 reduces arborization of sensory neurons in WAT and was associated with systemic insulin resistance, supporting adipocyte‐to‐sensory nerves communication [75]. Recent ablation experiments, ranging from selective sensory ablation to sensory mechanosensitive ion channel Piezo2 signaling deletion in WAT, have revealed that sensory innervation is involved in regulating WAT browning and acting as a brake on the sympathetic system [72, 76, 77]. In combination with these findings, our results raise the question of whether FSTL1 could also act on sensory innervation in WAT, which will remain to be further investigated. In addition to its possible adipocyte‐sensory nerves crosstalk, it also warrants further investigation into whether FSTL1 exerts a sensory neuron‐autonomous effect, considering its high expression levels in DRG neurons [57].

Several limitations of the current study should be acknowledged. In addition to the one mentioned above, while sympathetic cell lines were used in our in vitro experiments, primary sympathetic neurons will be required to validate FSTL1 as a neurotrophic factor. Second, future efforts will be required to perform the pharmacological FSTL1 approach in vivo. Given that systemic elevation of serum FTSL1 might be associated with hepatic fibrosis, pulmonary fibrosis, and other complications. It is of particular interest to investigate the molecular details of FSTL1‐TrkB binding to identify functional domains. Subsequently, we will explore targeted therapeutic approaches by developing peptide‐based compounds that selectively modulate TrkB in adipose tissue. Thirdly, haploinsufficiency of FSTL1 in preadipocytes was used to deplete FSTL1 within adipose tissue; it will be difficult to determine to which degree beige/brite progenitors contribute to impaired cold tolerance and WAT browning. Accordingly, the functions of FSTL1 on beige/brite progenitors are still not clear. Additionally, besides the endocytic trafficking mainly via the TrkB receptor, whether FSTL1 promotes sympathetic innervation via additional regulatory mechanisms remains undetermined. Lastly, the role of FSTL1 as a neurotrophic factor in regulating sensory nerves within adipose tissue remains to be elucidated.

## 4 Conclusion

Collectively, our study here, for the first time, reports the effect of FSTL1 on white fat browning. We confirm that FSTL1 induces the browning of white fat via promoting sympathetic nerve innervation. It is the first attempt to understand the paracrine manner of FSTL1 in adipose tissue. It is also the first attempt to confirm the anti‐obesity effect of FSTL1‐specific expression in adipose tissue. We further identify TrkB as the main receptor for FSTL1. The data indicate that FSTL1 is an adipose tissue‐derived ‘neurotrophic factor’ adipokine. The current study could potentially suggest FSTL1 as a therapeutic target in obesity and other metabolic disorders.

## 5 Methods

### 5.1 Animals

This study utilized adult male C57BL/6J mice aged 6–8 weeks weighing 20–25 g, unless otherwise indicated. Fstl1^flox/+^ mice were bred with Pdgfrα‐Cre mice to generate Fstl1^Pdgfrα+/−^ and Fstl1^Pdgfrα−/−^ mice. The mice were housed under a 12‐h light/12‐h dark cycle and provided ad libitum access to food and water. All animal experimental procedures followed the National Institutes of Health Guide for the Care and Use of Laboratory Animals and were approved by the Animal Use and Care Committee of Capital Medical University, Beijing, China (approval no. AEEI‐2024‐203). The study was conducted with great care for animal welfare, ensuring minimal use of animals while taking all necessary precautions to prevent any potential suffering.

For cold exposure experiments, mice were exposed to a 4°C environment for 7 days or at the indicated time points. For CL316,243 treatment experiments, mice were injected intraperitoneally with 1 mg/kg of CL316,243 (C5976; Sigma) for 7 days.

To achieve tissue‐specific overexpression, mice received local delivery of AAV2/8‐FSTL1 in adipose tissues. For specific overexpression in iWAT, mice were locally microinjected with AAV2/8‐FSTL1 (right) and AAV2/8‐EGFP (left) vectors into iWAT. For specific overexpression in iWAT and epididymal white adipose tissue (eWAT), mice received microinjections of AAV2/8‐FSTL1 into iWAT and eWAT on both sides. Mice receiving AAV2/8‐EGFP vectors microinjections were used as controls.

For prevention experiments, all mice were locally microinjected twice (1‐week interval) into bilateral adipose tissues. One group of mice received 6‐OHDA and AAV2/8‐FSTL1 injections (6‐OHDA+AAV2/8‐FSTL1) into iWAT and eWAT on both sides. Another group of mice received saline and AAV2/8‐FSTL1 injections (saline+AAV2/8‐FSTL1) into iWAT and eWAT on both sides. The third group of mice received saline and AAV2/8‐EGFP injections (saline+AAV2/8‐EGFP) into bilateral iWAT and eWAT.

For the ANA‐12 injection experiment, mice were divided into three groups. One group of mice received AAV2/8‐EGFP injections into iWAT on both sides. Another group of mice received AAV2/8‐ FSTL1 injections into iWAT on both sides. The third group of mice received AAV2/8‐ FSTL1 injections into bilateral iWAT, followed by intraperitoneal injections of ANA‐12.

The body weight, food intake, and water drinking were measured weekly. Additionally, the body temperature (rectal temperature) was also measured three times at room temperature or at the indicated times during cold exposure.

### 5.2 Adeno‐Associated Virus 2/8 Construction and Injection

AAV2/8 viral vectors carrying mouse FSTL1 and EGFP genes were produced by OBIO Technology Co Ltd (Shanghai, China). The FSTL1 gene expression was controlled by the CMV promoter and followed by Flag and EGFP genes. Adult male mice received injections of AAV2/8‐FSTL1 (5.56 × 10^10^ v.g/fat pad) or AAV2/8‐EGFP (5.56 × 1010 v.g/fat pad) throughout the fat pads.

### 5.3 Energy Metabolic Study

For analysis of energy metabolism, mice were placed in a metabolic cage (Oxylet, Panlab, Spain) for 24 h to acclimate to the environment. Subsequently, oxygen consumption (VO2) and carbon dioxide production (VCO2) were recorded every 30 min for 24 h. Respiratory quotient (RQ) was calculated as RQ = VCO2/VO2, and energy consumption (EE) was calculated as EE = (3.815 + 1.232 × RQ) × VO2 × 1.44 (unit: kcal/day/kg0.75) using Metabolism software. The food intake, water drinking, and motor activities were also monitored simultaneously by the equipment.

### 5.4 Glucose Tolerance and Insulin Tolerance Test

Glucose tolerance was assessed using an intraperitoneal glucose tolerance test (IPGTT). Mice were fasted for 16 h with free access to water and then given an intraperitoneal glucose injection (1.5 g/kg). Blood glucose levels were measured at 0, 15, 30, 60, and 120 min after injection using a NovaMax Glucometer. Insulin tolerance was assessed using an intraperitoneal insulin tolerance test (IPITT). Mice were fasted for 6 h with free access to water before IPITT. Insulin was administered intraperitoneally at 0.75 U/kg body weight. The levels of blood glucose were measured at 0, 15, 30, and 60 min using a NovaMax glucometer.

### 5.5 Small Animal PET/CT Experiments

Small animal PET/CT scanning was performed using a Siemens Inveon scanner. As previously described, [52] mice were placed in a prone position on the scanning bed after anesthesia with isoflurane. Glucose uptake was measured using 18 F‐FDG through 30‐min ambulatory acquisitions (1 × 30 s, 12 × 10 s, 8 × 30 s, 6 × 90 s, and 3 × 300 s) following tail vein injection of 18 F‐FDG (10‐12 MBq, 0.2 mL 0.9% NaCl). The levels of 18 F‐FDG uptake in iWAT, eWAT, tibialis anterior muscle, and triceps surae muscle were measured and expressed as standardized uptake values (SUV).

### 5.6 Oil Red O and Nile Red Staining

Oil Red O and Nile Red staining was performed on frozen sections with standard methods. Adipose tissues were fixed with cold 4% paraformaldehyde for 48 h and placed in 30% sucrose for 1 week. Then adipose tissue sections were cut at 25‐µm thickness on a frozen microtome. The slides were incubated in Oil Red O staining solution for 15 min at 60°C or in Nile Red staining solution for 10 min at 37°C. The Oil Red O‐stained slides were observed using a Nikon light microscope, and Nile Red‐stained slides were observed with a Leica fluorescence microscope.

### 5.7 Lentivirus Vectors Construction and Transduction

Lentiviral packaging and insertion of the coding sequence of genes were provided by OBIO Technology Co. Ltd. (Shanghai, China). The titers of lenti‐FSTL1 and lenti‐EGFP vectors were 2.96 × 108 and 2.76 × 108 TU/mL, respectively. For in vitro transduction experiments, both 3T3‐L1 and ADSCs were transduced with lenti‐FSTL1 (MOI: 10) or lenti‐EGFP (MOI: 10) in the presence of polybrene. After lentivirus transduction, the cells were selected with puromycin treatment for 14 days.

### 5.8 Cell Culture

3T3‐L1 cells were maintained in Dulbecco's Modified Eagle Medium (DMEM) supplemented with 10% Fetal Bovine Serum (FBS). For adipogenic differentiation, once cells reached confluence, culture medium was replaced with differentiation medium I (DMI, 5 µg/mL insulin, 0.5 mM 3‐isobutyl‐1‐methylxanthine, and 1 µM dexamethasone) for 2 days and followed by differentiation medium II (DMII, 5 µg/mL insulin) for another 2 days. Finally, the cells were cultured with DMEM with 10% FBS for 4 days. MN9D cells were cultured in F12 supplemented with 10% FBS. Then CM from transduced 3T3L1 cells (denoted as CM‐3T3L1^FSTL1^ and CM‐3T3‐L1^EGFP^, respectively) was collected, lypophilized, and stored for the following experiments. Additionally, CM from 3T3‐L1 cells without lentivirus transduction (denoted as CM‐3T3‐L1) was also collected for the next experiments. HEK‐293T was cultured in DMEM supplemented with 10% FBS and maintained at 37°C in an atmosphere of 5% CO_2_.

### 5.9 Isolation and Differentiation of Adipose‐Derived Stem Cells

As previously described [40], ADSCs were harvested from iWAT and eWAT of male mice, respectively. The ADSCs were cultured with DMEM/F12 containing 10% FBS. For adipogenic differentiation, ADSCs were maintained in DMI (5 µg/mL insulin, 0.5 mM 3‐isobutyl‐1‐methylxanthine, 1 µM dexamethasone, and 60 µM indomethacin) for 2 days and DMII (5 µg/mL insulin) for another 2 days. Then the cells were cultured with DMEM/F12 with 10% FBS for 4 days.

### 5.10 Cell Proliferation Assay

Cell proliferation ability was assessed with a Cell Counting Kit‐8 kit according to the manufacturer's instructions. MN9D cells were seeded into a 96‐well plate at a density of 5000 cells per well. The cells were incubated with rFSTL1 (100 ng/mL), and cell proliferation ability was determined at the indicated time points.

### 5.11 Enzyme‐Linked Immunosorbent Assay

The protein levels of FSTL1 in CM were determined with a mouse FSTL1 Enzyme‐linked immunosorbent assay (ELISA) Kit (Cloud‐Clone Corp, Wuhan, China) according to the manufacturer's instructions. The NE levels from MN9D culture media were detected with a mouse NE ELISA kit (MLbio, Shanghai, China) according to the manufacturer's instructions. The T‐CHO, TG, NEFA, ALT, and AST in serum were detected with a mouse T‐CHO, TG, NEFA, ALT, and AST ELISA kits (Jiancheng Corp, Nanjing, China) according to the manufacturer's instructions.

### 5.12 Sympathetic Denervation

Sympathetic denervation of iWAT and eWAT was performed as previously described [78, 79]. Neurotoxin 6‐OHDA (MCE, HY‐B1081A), which selectively destroys sympathetic nerves, was dissolved in a 0.9% NaCl solution containing 0.2% sodium ascorbate. Adult male mice received microinjections of 6‐OHDA into multiple points of iWAT and eWAT at a 16 µg/pad dosage. After 6‐OHDA injections, mice were allowed to recover for 1 week and then received AAV2/8 vector injections.

### 5.13 Western Blotting

Proteins were extracted from tissues or cells using a RIPA kit (Beyotime, Shanghai, China) following the instructions. Cytoplasmic, nuclear, and membrane proteins were extracted following the manufacturer's protocol (Cat No. P0028, Cat No. P0033, Beyotime Biotechnology, Shanghai, China). The proteins were then separated using 10%–12% SDS‐PAGE and transferred onto PVDF membranes. The membranes were incubated with TBST containing 5% skimmed milk or 5% BSA, followed by primary and secondary antibodies. The primary antibodies used were anti‐UCP1 (1:1000, ab10983, Abcam), anti‐FSTL1 (1:1000, AF1738, Rr&D), anti‐PPAR‐γ (1:1000, 2430, Cell Signaling Technology), anti‐TH (1:1000, #58844, Cell Signaling Technology), anti‐β‐actin (1:2000, ab8227, Abcam), anti‐Flag (1:1000 ab1170, Abcam), rabbit anti‐His (1:5000, 66005‐1, Proteintech), anti‐NT‐4 (1:1000, Proteintech), anti‐BDNF (1:1000, Proteintech), anti‐NT‐3 (1:1000, Proteintech), anti‐phospho‐PKCα (Thr638) (1:1000, Proteintech), anti‐pan‐PKC (1:1000, Proteintech), anti‐His (1:2000, Proteintech), anti‐TrkB (1:1000, Proteintech, 13129‐1‐AP), anti‐p‐AKT1/2/3 (1:1000, Abcam, ab131443), anti‐AKT1/2/3 (1:2000, Abcam, ab179463), anti‐ERK1/2 (1:1000, CST, 4695S), anti‐p‐ERK1/2 (Thr202/Tyr204) (1:2000, CST, 4370S), and anti‐Histone H3 (1:1000, Beyotime, AF0009). GAPDH, β‐actin, and β‐tubulin were used as loading controls. The bands were quantified using ImageJ software (NIH, Bethesda, MD, USA). The protein amounts were normalized to that of GAPDH, β‐actin, or β‐tubulin.

### 5.14 Quantitative Real‐Time PCR

Total RNA was extracted from tissues and cells using the Total RNA Extractor (Trizol) (Sangon Biotech, Beijing, China) following the manufacturer's guidelines. The cDNA was then synthesized from the extracted RNA following the instructions provided in the HiScript II First Strand cDNA Synthesis Kit (Vazyme, Jiangsu, China). Quantitative real‐time polymerase chain reaction was performed using ChamQ SYBR qPCR Master Mix (Vazyme, Jiangsu, China) in a final volume of 20 µL containing 10 µM of both forward and reverse primers. The mRNA results in each sample were standardized using the housekeeping gene β‐actin as an internal control. The primer sequences can be found in supplementary Table S1. The CFX96 Real‐time System and C1000 Thermal Cycler (BioRad) were used to quantify relative mRNA levels.

### 5.15 Histological Analysis

The adipose tissue and liver were fixed with 4% paraformaldehyde and preserved through paraffin embedding or cryopreservation. Paraffin tissues were cut into 6‐µm thick sections and frozen tissues were cut into 25‐µm thick sections, respectively. Hematoxylin and Eosin (HE) staining was performed according to standard methods. For immunohistochemistry (IHC) staining, the primary antibody was rabbit anti‐UCP1 (1:100, ab10983, Abcam), and the secondary antibody was goat anti‐rabbit IgG.

For immunofluorescent staining, the primary antibodies were rabbit anti‐UCP1 (1:100, ab10983, Abcam), rat anti‐FSTL1 (1:400, MAB17381, R&D), and rabbit βIII‐tubulin (1:500, T2200, Sigma). The secondary antibody was Alexa Fluor‐594‐conjugated goat anti‐rabbit IgG (1:1000, Invitrogen). For double‐label immunofluorescent staining, the primary antibodies were guinea pig anti‐Perilipin1 (1:250, GP29, Progen), chicken anti‐EGFP (1:1000, ab13970, Abcam), rabbit anti‐UCP1 (1:100, ab10983, Abcam), and rabbit anti‐TH (1:200, ab75875, Abcam), rabbit anti‐TrkB (1:200, Proteintech, 13129‐1‐AP), and mouse anti‐His (1:400, 66005‐1, Proteintech). The secondary antibodies were Rhodamine Red‐X‐conjugated goat anti‐guinea pig IgG (1:200, Jackson Immunoresearch Laboratories), Alexa Fluor‐488‐conjugated goat anti‐chicken IgG (1:200, Jackson Immunoresearch Laboratories), Alexa Fluor‐594‐conjugated goat anti‐mouse IgG (1:200, Jackson Immunoresearch Laboratories), Alexa Fluor‐488‐conjugated goat anti‐rabbit IgG (1:200, Jackson Immunoresearch Laboratories), and Alexa Fluor‐594‐conjugated goat anti‐rabbit IgG (1:1000, invitrogen). Membrane staining was used with DiO according to the manufacturer's instructions (C1038, Beyotime, China). The sections were examined by a Nikon light microscope or Leica fluorescence microscope.

For Masson staining, the sections were stained using the corresponding Masson trichome staining kits (Solarbio Science & Technology Co., Ltd, Beijing) according to the manufacturer's instructions.

### 5.16 Whole Mount Clearing

The whole‐mount adipose tissues were processed with the Adipo‐Clear to allow immunostaining and three‐dimensional visualization, which was supported by Nuohai Life Science Co., Ltd. (Shanghai, China). Similar to the previous description [43], adipose tissues were harvested after intracardiac perfusion and post‐fixed with 4% PFA at 4°C overnight. The fixed samples were processed through serial steps including delipidation, permeabilization, immunostaining, clearing, three‐dimensional image reconstruction, and image processing. The primary antibodies were rabbit anti‐TH (1:1000, AB152, Millipore) and chicken anti‐Flag (1:200, ab1170, Abcam). The secondary antibodies were Alexa Fluor‐488‐conjugated goat anti‐chicken IgG (1:200, Jackson Immunoresearch Laboratories) and Alexa Fluor‐594‐conjugated goat anti‐rabbit IgG (1:1000, Invitrogen). The image was obtained with ImspectorPro software (LaVision BioTec), and the image processing was conducted with Imaris x64 software (version 8.0.1, Bitplane).

### 5.17 Molecular Docking Analysis

The interaction between the FSTL1 and TrkB proteins was performed utilizing computational structural simulation methodologies. The TrkB structure was anticipated using SWISS‐MODEL (https://swissmodel.expasy.org/). The FSTL1 structure was obtained from RCSB (https://www.rcsb.org/). Subsequently, the anticipated TrkB structure was aligned with the FSTL1 protein configuration (PDB accession number: 6JZA) through the ZDOCK Server (https://zdock.wenglab.org/). Ultimately, PyMOL was employed to visualize the docking schematic.

### 5.18 Co‐Immunoprecipitation Assays

Interactions between FSTL1 and TrkB were determined by Co‐IP. 293T cells were lysed with IP lysis buffer containing a protease inhibitor cocktail on ice for 30 min. After centrifugation at 12 000 g for 15 min at 4°C, lysates were incubated with anti‐Flag antibody at 4°C overnight. Protein A/G Magnetic Beads (MCE, HY‐12028) were later added to the mixtures at 4°C for 3 h. Subsequently, after three washes with IP lysis, the coprecipitated proteins were obtained and analyzed by Western blotting.

### 5.19 RNA Interference

siRNA transfections were performed using Lipofactamine RNAi MAX according to the manufacturer's protocol, with a final siRNA concentration of 10 nM. Cells were harvested at 72–96 h post‐transfection, depending on the experimental objective. For target genes, at least three independent siRNA sequences were selected, and the sequences with the highest knockdown efficiency were used for subsequent studies. TrkC siRNA is obtained from Santa Cruz Biotechnology (Cat. No. sc‐36730).

### 5.20 Statistical Analysis

Metabolic cage data from the first 24 h were excluded to allow acclimation, and energy expenditure was normalized to body weight. Western blot and qPCR signals were normalized to internal control and expressed as fold‐change relative to the control group prior to statistical analysis. All data are presented as the mean ± standard error of the mean (SEM). Statistical analyses were performed using GraphPad Prism 8.0. A one‐way analysis of variance (ANOVA) followed by Tukey's test was performed for comparisons among multiple groups. A two‐way repeated‐measures ANOVA was performed for time‐course measurements, including IPGTT, IPITT, body weight monitoring, and body temperature after cold exposure. An unpaired Student's *t*‐test was performed for comparisons between two groups. Statistical significance was shown as ^*^ *p* < 0.05, ^**^ *p* < 0.01, or ^***^ *p* < 0.001.

## Author Contributions

X.W.J. and J.X.L. performed the experiments; R.P.W., X.Q.W., Y.C., M.C.W. and Y.X. provided help in cell culture experiments and animal experiments; H.W.S. and X.L. provided help in staining; T.L. provided assistance on data analysis; L.L.W., and C.Y.J. contributed to data analysis and review/edits on manuscript; D.L.F., C.Y. and Y.G. conceived, designed the study and wrote the manuscript.

## Conflicts of Interest

The authors declare no conflicts of interest.

## Supporting information




**Supporting File 1**: advs77433‐sup‐0001‐SuppMat.docx.

**Supporting File 2**: advs77433‐sup‐0002‐VideoS1.mp4.

## Data Availability

Data sharing not applicable to this article as no datasets were generated or analysed during the current study.
