# Supplementary material for: The Adipose‐Sympathetic Nerve Crosstalk: FSTL1 as an Adipocyte‐Derived Neurotrophic Factor for WAT Browning
Source: Adv Sci (Weinh). 2026 Aug 31:e77433. Online ahead of print. doi: 10.1002/advs.77433 (PMC13530021; doi:10.1002/advs.77433)
Supplement: Supplementary file 1 [file ADVS-9999-e77433-s002.docx]

**The Adipose-Sympathetic Nerve Crosstalk: FSTL1 as an Adipocyte-Derived Neurotrophic Factor for WAT Browning**

Xiao-Wei Jia ^a,^ †, Jia-Xin Liu ^a,^ †, Rui-Ping Wang ^a^, Xue-Qin Wu ^a^, Yao Che ^a^, Mu-Chen Wu ^a^, Hong-Wei Shang ^b^, Xin Lu ^b^, Tao Lu ^a^, Lu-Lu Wang ^a^, Chun-Yan Jiang ^c^, Dong-Liang Fang ^a,^ ***, Chun Yang ^a c,^ **, Yan Gao ^a, d,^ *

a Department of Human Anatomy, School of Basic Medical Sciences, Capital Medical University, Beijing 100069, China.

b Department of National Experimental Center for Basic Medical Teaching, School of Basic Medical Sciences, Capital Medical University, Beijing 100069, China.

c Sports medicine Department, Beijing Jishuitan Hospital, Capital Medical University, Beijing 102208, China.

d Key Laboratory of Major Diseases in Children, Ministry of Education, China.

*** Correspondence to Dong-liang Fang, Department of Human Anatomy, Capital Medical University, No. 10 Xitoutiao, You’anmenwai, Fengtai District, Beijing 100069, China. E-mail address: sdwffdl@163.com.

** Correspondence to Chun Yang, Department of Human Anatomy, Capital Medical University, No. 10 Xitoutiao, You’anmenwai, Fengtai District, Beijing 100069, China. E-mail address: yangchunsjz@126.com.

* Correspondence to Yan Gao, Department of Human Anatomy, Capital Medical University, No. 10 Xitoutiao, You’anmenwai, Fengtai District, Beijing 100069, China. E-mail address: gy1003@ccmu.edu.cn. Tel: +8601083950485

† These authors contributed equally.

**Supplementary table**

**Table S1: The prime sequences of quantitative Real-time PCR**

| **Name** | **Forward/Reverse** | **Sequence (5'to3')** |
| --- | --- | --- |
| *Ucp1* | Forward primer | ACTGCCACACCTCCAGTCATT |
|  | Reverse primer | CTTTGCCTCACTCAGGATTGG |
| *Fstl1* | Forward primer | CTATCAAGCTAACCGCGAT |
|  | Reverse primer | GTCGCCATTATCAAAGCTCT |
| *Ppargc1a* | Forward primer | AGCCGTGACCACTGACAACGAG |
|  | Reverse primer | GCTGCATGGTTCTGAGTGCTAAG |
| *Cd137* | Forward primer | CGTGCAGAACTCCTGTGATAAC |
|  | Reverse primer | GTCCACCTATGCTGGAGAAGG |
| *Prdm16* | Forward primer | CCACCAGCGAGGACTTCAC |
|  | Reverse primer | GGAGGACTCTCGTAGCTCGAA |
| *Tmem26* | Forward primer | GGCACCATCACTAGAGACC |
|  | Reverse primer | CAGCATACTCCACGTCCACA |
| *Cebpa* | Forward primer | CCGTGGTGGTTTCTCCTTGA |
|  | Reverse primer | TTTTTGCTCCCCCTACTCGG |
| *Pparg* | Forward primer | GAAGAACCATCCGATTGAAGC |
|  | Reverse primer | AAGGCACTTCTGAAACCGACA |
| *Fabp4* | Forward primer | TTTACAAAATGTGTGATGCCTT |
|  | Reverse primer | TAAAAGTACTCTCTGACCGGAT |
| *Adipoq* | Forward primer | GACTAATGAGACCTGGCCACT |
|  | Reverse primer | AGTAGCATCCTGAGCCCTTTT |
| *Cidea* | Forward primer | TGCTCTTCTGTATCGCCCAGT |
|  | Reverse primer | GCCGTGTTAAGGAATCTGCTG |
| *Dio2* | Forward primer | CAGTGTGGTGCACGTCTCCAATC |
|  | Reverse primer | TGAACCAAAGTTGACCACCAG |
| *Il6* | Forward primer | GGATACCACTCCCAACAGACC |
|  | Reverse primer | GCAAGTGCATCATCGTTGTTC |
| *Il1b* | Forward primer | AATCTCGCAGCAGCACATCAAC |
|  | Reverse primer | TGTCCTCATCCTGGAAGGTC |
| *Adgre1* | Forward primer | CTTTGGCTATGGGCTTCCAGTC |
|  | Reverse primer | GCAAGGAGGACAGAGTTTATCGTG |
| *Cd34* | Forward primer | ATCCCCATCAGTTCCTACCAAT |
|  | Reverse primer | TGGTGTGGTCTTACTGCTGTC |
| *Ccl2* | Forward primer | CAGCAGGTGTCCCAAAGAAGC |
|  | Reverse primer | TGTCTGGACCCATTCCTTCTTG |
| *Tnfa* | Forward primer | CTCATCAGTTCTATGGCCCAG |
|  | Reverse primer | GTAGACAAGGTACAACCCATCG |
| *Acta2* | Forward primer | CCCAGACATCAGGGAGTAATGG |
|  | Reverse primer | TCTATCGGATACTTCAGCGTCA |
| *Col1a1* | Forward primer | TGCTAACGTGGTTCGTGACCGT |
|  | Reverse primer | ACATCTTGAGGTCGCGGCATGT |
| *Col6a1* | Forward primer | AACAGGAATAGGAAATGTGACCC |
|  | Reverse primer | ACACCACGGATAGGTTAGGGG |
| *Tgfb1* | Forward primer | ATTTGGAGCCTGGACACACA |
|  | Reverse primer | GAGCGCACAATCATGTTGGA |
| *Hif1a* | Forward primer | CAAGATCTCGGCGAAGCAA |
|  | Reverse primer | CTGTCTAGACCACCGGCATC |
| *Cox7a1* | Forward primer | GCTCTGGTCCGGTCTTTTAGC |
|  | Reverse primer | GTACTGGGAGGTCATTGTCGG |
| *Cox8b* | Forward primer | GAACCATGAAGCCAACGACT |
|  | Reverse primer | GCGAAGTTCACAGTGGTTCC |
| *Atpb* | Forward primer | GACATGGGCACAATGCAGG |
|  | Reverse primer | GCAGGGTCAGTCAGGTCATCA |
| *Chchd3* | Forward primer | GCGGACGAGAACGAGAACAT |
|  | Reverse primer | TCGCTGAGACTTAGAGCCAGA |
| *Chchd10* | Forward primer | CAGCCGGGTCTTATGGCTC |
|  | Reverse primer | CAGGCTCTGAATTTCCCCCAC |
| *Ppargc1b* | Forward primer | TCCTGTAAAAGCCCGGAGTAT |
|  | Reverse primer | GCTCTGGTAGGGGCAGTGA |
| *Sdhd* | Forward primer | AATTTGCCATTTACCGATGGGA |
|  | Reverse primer | AGCATCCAACACCATAGGTCC |
| *Idh3b* | Forward primer | TGGAGAGGTCTCGGAACATCT |
|  | Reverse primer | AGCCTTGAACACTTCCTTGAC |
| *Ogdh* | Forward primer | GTTTCTTCAAACGTGGGGTTCT |
|  | Reverse primer | GCATGATTCCAGGGGTCTCAAA |
| *Sucla2* | Forward primer | ACCCTTTCGCTGCATGAATAC |
|  | Reverse primer | CCTGTGCCTTTATCACAACATCC |
| *Ndufa6* | Forward primer | TCGGTGAAGCCCATTTTCAGT |
|  | Reverse primer | CTCGGACTTTATCCCGTCCTT |
| *Th* | Forward primer | CCAAGGTTCATTGGACGGC |
|  | Reverse primer | CTCTCCTCGAATACCACAGCC |
| *Ntrk2* | Forward primer | GTCAGCCCTCACGTCACTTC |
|  | Reverse primer | CAACTGCGGTAGCAGGACA |
| *Bdnf* | Forward primer | AATTGGCTGGCGATTCATAAGG |
|  | Reverse primer | AAGTTGTGCGCAAATGACTGTT |
| *Ntf4* | Forward primer | GGATTCGGATCGACACAGCTT |
|  | Reverse primer | GTCATAAGGCACGGTAGAGCA |
| *Ntf3* | Forward primer | GCGCTGGATACGAATAGACACT |
|  | Reverse primer | CGAGAGCCCAATCACAAAACAA |

**Supplementary Figures**

**Figure S1. FSTL1 correlates with UCP1 expression in white adipose tissue browning.** (A) Representative images of HE sections and UCP1 immunohistochemical staining of iWAT from wild-type mice following one week of cold exposure or CL316,243 injections (n = 3 per group). Scale bars = 50 μm. (B) Relative mRNA levels of *Ucp1* and *Fstl1*in iWAT as in (A) (n = 3 per group). (C) Immunoblot and quantification of UCP1 and FSTL1 of iWAT in (A) (n = 3 per group). (D, E) Representative images show FSTL1 (D) and UCP1 (E) immunofluorescence staining of iWAT in (A) (n = 3 per group). Scale bars = 75 μm. Data are presented as mean ± SEM. A one-way ANOVA followed by a Tukey’s test was used to make group comparisons. *p < 0.05, **p < 0.01, ***p < 0.001 versus control group.

**Figure S2. FSTL1 overexpression in iWAT improves metabolic homeostasis in mice with diet induced obesity.** (A) The graph showing the body weight of mice with HFD feeding for 12 weeks and followed by local delivery of AAV2/8-FSTL1 or AAV2/8-EGFP vectors (n = 6 per group). (B, C) The graphs showing blood glucose at indicated time points after glucose (B) and insulin (C) injections of mice as in (A) (n = 6 per group). (D) Representative macroscopy images and weight quantification of iWAT and eWAT from mice as in (A) (n = 5 per group). (E, F) Representative HE staining and the diagram showing the adipocytes size of iWAT and eWAT from mice as in (A) (n = 5 per group). (G-I) Representative images of UCP1 immunohistochemical staining (G), as well as the immunoblot (H) and quantification (I) of UCP1 and FSTL1 in iWAT as in (A) (n = 4 per group). (J, K) Relative mRNA levels of adipogenesis, thermogenesis (J), inflammation, and fibrosis genes (K) in iWAT from mice as in (A) (n = 5 per group). (L) The graph showing the concentration of total cholesterol (T-CHO), triglycerides (TG), and non-esterified free fatty acids (NEFA) in serum of mice as in (A) (n = 5 per group). (M) Representative HE staining images and weight of liver from mice as in (A) (n = 5 per group). (N) The graphs showing the concentration of AST and ALT in serum of mice as in (A) (n = 5 per group). (O, P) Representative Masson staining and relative mRNA levels of inflammation, and fibrosis genes in liver of mice as in (A) (n = 5 per group). (Q) The diagram showing FSTL1 protein levels in serum of mice as in (A) (n = 5 per group). Data are presented as mean ± SEM. A two-way repeated-measures ANOVA was used for group comparison in (A)-(C). An unpaired Student’s *t*-test was used to make group comparisons (D)-(Q). *p < 0.05, **p < 0.01, ***p < 0.001 versus HFD+AAV2/8-EGFP group.

**Figure S3. Haploinsufficiency of FSTL1 in preadipocytes leads to impaired cold-induced thermogenesis and exacerbates the effects of diet-induced obesity (DIO).** (A) Genotyping confirms the presence of *Fstl1^Pdgfrα+/+^* mice (samples 5, 6, 7, 8, 11, 12, and 13) and *Fstl1^Pdgfrα+/-^* mice (samples 2, 10, and 14). (B) Immunohistochemical staining sections of UCP1 of eWAT from *Fstl1^Pdgfrα+/+^* and *Fstl1^Pdgfrα+/-^* mice with cold exposure. (C) Weights of eWAT from the mice as in (B) (n = 6 per group). (D) Immunoblot and quantification of FSTL1 and UCP1 in eWAT from the mice as in (B) (n = 6 per group). (E, F) Relative mRNA expression levels of adipogenesis, thermogenesis, and mitochondria-related genes in eWAT from the mice as in (B) (n = 6 per group). (G) Images showing UCP1 immunostaining in BAT of mice as in (B). (H) Weight of BAT of mice as in (B) (n = 6 per group). (I) Immunoblot and quantification of BAT from mice as in (B) (n = 4 per group). (J, K) Relative mRNA expression levels of adipogenesis, and mitochondria-related genes in BAT from the mice as in (B) (n = 6 per group). (L) Representative images of HE-stained sections of iWAT, eWAT, BAT, and liver from *Fstl1^Pdgfrα+/+^* and *Fstl1^Pdgfrα+/-^* mice fed an HFD. Data are presented as mean ± SEM. An unpaired Student’s *t*-test was used to make group comparisons. *p < 0.05, **p < 0.01, ***p < 0.001 versus *Fstl1^Pdgfrα+/+^* group.

**Figure S4. FSTL1 cannot increase the expression of browning related genes *in vitro*.** (A-F) Immunoblot and quantification of UCP1 protein (A, C, E) (n = 3 per group), and relative mRNA levels of browning related genes (*Ucp1, Ppargc1a, Prdm16, Tmem26, CD137*, and *Cidea*) (B, D, F) (n = 5 per group) in ADSCs from iWAT (A, B), ADSCs from eWAT (C, D), and 3T3L1 cell lines (E, F) after rFSTL1 treatments. (G-L) Immunoblot of UCP1 protein (G, I, K) (n = 3 per group), and relative mRNA levels of browning related genes (*Ucp1, Ppargc1a, Prdm16, Tmem26, CD137, and Cidea*) (H, J, L) (n = 5 per group) in ADSCs from iWAT (G, H), ADSCs from eWAT (I, J), and 3T3L1 cell lines (K, L) with FSTL1 overexpression. Data are presented as mean ± SEM. A one-way ANOVA followed by a Tukey’s test was used to perform group comparisons. NS, not significant. **p < 0.01, ***p < 0.001 versus indicated group.

**Fig. S5. FSTL1 overexpression in iWAT leads to elevated TH levels.** (A, B) Immunoblot and quantification of TH protein in iWAT of HFD+AAV2/8-EGFP and HFD+AAV2/8-FSTL1 groups. (C, D) Immunoblot and quantification of relative TH levels in the AAV2/8-EGFP+HFD and AAV2/8-FSTL1+HFD groups. Data are presented as mean ± SEM. n = 4 per group. An unpaired Student’s *t*-test was used to perform group comparisons. **p < 0.01, ***p < 0.001 versus indicated group.

**Figure S6. The body weight, food intake, water drinking, and activity of mice.** (A) The body weight of mice with 6-OHDA treatments, followed by local delivery of AAV2/8-FSTL1 or AAV2/8-EGFP under room temperature. (B-D) The graphs show food intake (B), water drinking (C), and activity (D) of mice in (A). Data are presented as mean ± SEM. n = 6 per group. A one-way ANOVA followed by a Tukey’s test was used to perform group comparisons. NS, not significant versus indicated group.

Figure S7. The localization of rFSTL1 and downstream signaling pathways in MN9D cells following FSTL1 treatments. (A) Double fluorescent immunostaining showing the colocalization of FSTL1 and TrkB in MN9D cells with rFSTL1-His treatment. (B) Immunoblot analysis of rFSTL-His protein in cytosol and nucleus of MN9D cells with rFSTL1-His treatment. (C) Representative blotting showing downstream signaling pathways in MN9D cells with rFSTL1-His treatment. (D) The diagram showing the relative TrkB mRNA levels in n MN9D cells with rFSTL1-His treatment. Data are presented as mean ± SEM. n = 3 per group. Data are presented as mean ± SEM.. An unpaired Student’s *t*-test was used to perform group comparisons. NS，no significant.

Figure S8. The effect of FSTL1 on TrkB ligands expression in adipose tissue. (A-B) The diagrams showing relative mRNA levels of BDNF, NT3, and NT4 in adipose tissue with FSTL1 overexpression with chow diet (A) or HFD (B). (C) The relative mRNA levels of BDNF, NT3, and NT4 in adipose tissue with FSTL1 deficicency. Data are presented as mean ± SEM. n = 6 per group. An unpaired Student’s *t*-test was used to perform group comparisons. NS，no significant.
